# Distinct responses of growth and respiration to growth temperatures in two mangrove species

**DOI:** 10.1093/aob/mcab117

**Published:** 2021-09-11

**Authors:** Tomomi Inoue, Yasuaki Akaji, Ko Noguchi

**Affiliations:** National Institute for Environmental Studies, 16-2 Onogawa, Tsukuba, Ibaraki, Japan; National Institute for Environmental Studies, 16-2 Onogawa, Tsukuba, Ibaraki, Japan; School of Life Sciences, Tokyo University of Pharmacy and Life Sciences, 1432-1 Horinouchi Hachioji, Tokyo, Japan

**Keywords:** Acclimation, growth, mangroves, O_2_ respiration, respiration cost, temperature

## Abstract

**Background and Aims:**

Mangrove plants are mostly found in tropical and sub-tropical tidal flats, and their limited distribution may be related to their responses to growth temperatures. However, the mechanisms underlying these responses have not been clarified. Here, we measured the dependencies of the growth parameters and respiration rates of leaves and roots on growth temperatures in typical mangrove species.

**Methods:**

We grew two typical species of Indo-Pacific mangroves, *Bruguiera gymnorrhiza* and *Rhizophora stylosa*, at four different temperatures (15, 20, 25 and 30 °C) by irrigating with fresh water containing nutrients, and we measured growth parameters, chemical composition, and leaf and root O_2_ respiration rates. We then estimated the construction costs of leaves and roots and the respiration rates required for maintenance and growth.

**Key Results:**

The relative growth rates of both species increased with growth temperature due to changes in physiological parameters such as net assimilation rate and respiration rate rather than to changes in structural parameters such as leaf area ratio. Both species required a threshold temperature for growth (12.2 °C in *B. gymnorrhiza* and 18.1 °C in *R. stylosa*). At the low growth temperature, root nitrogen uptake rate was lower in *R. stylosa* than in *B. gymnorrhiza*, leading to a slower growth rate in *R. stylosa*. This indicates that *R. stylosa* is more sensitive than *B. gymnorrhiza* to low temperature.

**Conclusions:**

Our results suggest that the mangrove species require a certain warm temperature to ensure respiration rates sufficient for maintenance and growth, particularly in roots. The underground temperature probably limits their growth under the low-temperature condition. The lower sensitivity of *B. gymnorrhiza* to low temperature shows its potential to adapt to a wider habitat temperature range than *R. stylosa*. These growth and respiratory features may explain the distribution patterns of the two mangrove species.

## INTRODUCTION

Mangrove plants are mostly found in tropical and sub-tropical tidal flats (Tomlinson, 1986; Spalding *et al.*, 2010). The particularity of their habitats has drawn much interest from plant biologists. On the basis of their global distribution pattern, Duke *et al.* (1998) suggested that mangroves occur in habitats where the mean winter temperature of the water surface is >20 °C. Instantaneous extreme freezing events may determine the latitudinal range limit of the mangrove *Avicennia germinans* (L.) L. in coastal Louisiana, USA (Pickens and Hester, 2011; Osland *et al*., 2014, 2015, 2017). The speed at which cultivated seedlings of *Rhizophora stylosa* Griff. grow increases as the temperature rises from 15 to 30 °C, and their photosystem II is photoinhibited at temperatures <20 °C (Akaji *et al.*, 2019). Although a warm temperature (almost above 20 °C) is clearly needed for the maintenance and growth of mangrove plants, the mechanisms underlying these phenomena have not been clarified.

Respiratory ATP is used for maintenance and growth requirements such as protein turnover, maintenance of solute concentration gradients across membranes, synthesis of new structures, nutrient uptake, phloem loading and nitrogen (N) assimilation in plants (de Wit *et al.*, 1970; Amthor, 1989; Thornley and Johnson, 1990; O’Leary *et al.*, 2019). Growth temperature affects the maintenance and growth processes and respiratory O_2_ consumption. Therefore, growth and respiration responses to temperature are closely related to each other. For example, changes in root temperature affect the rates of respiration and nutrient uptake in the roots, and whole-plant growth and nutrient demand change to adjust to the nutrient uptake capacity (Chapin, 1974; Chapin and Bloom, 1976; Clarkson *et al.*, 1992). Therefore, clarification of the temperature dependencies of respiration rates in mangroves and their link to growth properties would improve our understanding of the temperature limitation of growth of mangrove plants and how they acclimate to various growth temperatures.

Responses of respiration rates to changing temperatures vary among plant functional types and among their biomes. Meta-analyses of leaf CO_2_ respiration rates (i.e. CO_2_ emission rates) suggest that these rates under growing temperature are higher in plants from warm climates than in those from cold climates (Wright *et al.*, 2005; Atkin *et al.*, 2015). The warm habitats of mangroves raise the question of whether these plants require a comparatively high respiratory ATP (i.e. high O_2_ respiration rate) for their maintenance and growth. Previous studies reported that leaf and root respiration rates of mangrove species in the genera *Avicennia*, *Bruguiera* and *Rhizophora* lie within the range of 1–6 nmol O_2_ or CO_2_ g^−1^ s^−1^ (Burchett *et al.*, 1984; Hovenden *et al.*, 1995; Lovelock *et al.*, 2006; Atreya and Bhargava, 2008). This range is lower than that of terrestrial plants, i.e. 2–16 nmol O_2_ g^−1^ s^−1^ (Zha *et al.*, 2001; van Iersel, 2006; Laureano *et al*., 2008, 2013; Hernández-Montes *et al.*, 2019). Relative growth rates (RGRs) of mangrove plants range from 2 to 6 mg g^−1^ d^−1^ under fresh or saline water conditions (Lin and Sternberg, 1993; Farnsworth *et al.*, 1996; Ball *et al.*, 1997; Ellison and Farnstworth, 1997; Lόpez-Hoffman *et al.*, 2006). Because RGRs are 100–400 mg g^−1^ d^−1^ in herbaceous species and 10–150 mg g^−1^ d^−1^ in terrestrial tree species (Poorter and Garnier, 2007), the RGRs of mangrove plants are lower than those of other plants even under non-saline conditions. Even though low rates of growth and respiration appear to be characteristics of mangrove plants, the relationship between growth and respiration rates and their dependencies on growth temperatures are still unknown. Furthermore, most of the above studies on mangroves examined either leaf or root respiration rates, whereas both organs should be analysed to relate growth and maintenance responses to changing temperature. Previously, we found that temperature dependencies of CO_2_ respiration rates of stems and roots differed from those of leaves in *R. stylosa*, suggesting that responses of the CO_2_ respiration rate to long-term changes in growth temperature may differ between non-assimilatory and assimilatory organs in this species (Akaji *et al.*, 2019).

Here, we focused on two typical Indo-Pacific mangrove species, *Bruguiera gymnorrhiza* (L.) Lam. and *R. stylosa*. Both belong to the family Rhizophoraceae, but the former grows in a wide range of western Indo-Pacific areas, whereas the latter does not grow in eastern and southern Africa or in the Middle East (Spalding *et al.*, 2010). The temperature range of the habitats is wider in *B. gymnorrhiza* than in *R. stylosa* (Supplementary data Fig. S1), suggesting differences in the temperature dependencies of their growth and respiration rates.

We aimed to compare the dependencies of the growth parameters and O_2_ respiration rates of leaves and roots on growth temperatures in these two species. We hypothesized that: (1) the two mangrove species have adapted to warm conditions to ensure an O_2_ respiration rate sufficient for their maintenance and growth; (2) temperature dependencies of growth parameters and O_2_ respiration rate are linked between leaves and roots; (3) temperature dependencies of growth parameters and O_2_ respiration rate differ between the two species; and (4) *B. gymnorrhiza* has a wider potential range of growth temperatures than *R. stylosa*.

We grew seedlings at four temperatures, measured growth parameters, chemical compositions, and leaf and root O_2_ respiration rates, and estimated the construction costs and respiration rates for maintenance and growth. We examined the relationships between growth parameters and O_2_ respiration rates, and discuss the implications in terms of warm habitats of the mangrove species.

## MATERIALS AND METHODS

### Plant materials and growth conditions

For several months each year, *B. gymnorrhiza* and *R. stylosa* produce viviparous seedlings (diaspores) with extended hypocotyls; these diaspores drop from the mother trees when the diaspore length becomes 20–25 cm. In this study, diaspores of *B. gymnorrhiza* and *R. stylosa* (200 of each) were collected from at least 30 mature trees on Iriomote Island (24°17′33′′N, 123°51′43′′E), one of the southernmost islands of Japan, during the fruiting season (18 January 2018 for *B. gymnorrhiza* and 12 July 2017 for *R. stylosa*). According to the Japan Meteorological Agency, the mean monthly precipitation and temperature was 220 mm and 28.0 °C, respectively, in summer (from July to August), and 159 mm and 19.2 °C, respectively, in winter (from November to February) (https://www.data.jma.go.jp/obd/stats/data/mdrr/index.html). The collected diaspores were planted in a tray filled with sand (Imai Co. Ltd, Tsukuba, Japan) in a glasshouse (air temperature, 25 °C; relative humidity, 70 %) and watered twice a day. After about 6 months, 100 seedlings were randomly selected and transplanted individually into polycarbonate pots (159 mm diameter; 246 mm depth) with sand. From that time point, we started fertilization with 1:2000-diluted nutrient solution (NO_3_^–^, 2.34 mm; NH_4_^+^, 0.62 mm; PO_4_^–^, 0.50 mm; K, 2.86 mm; Ca, 1.04 mm; Na, 0.13 mm; Mg, 0.42 mm; Fe, 0.01 mm) (Hyponex Japan, Osaka, Japan) twice a day until the end of the cultivation period. There was a drain hole at the bottom of the pots, and 1 L of nutrient solution was showered on each seedling per day. A week after transplantation, 48 seedlings were randomly selected, and 12 seedlings of each species were transferred into four sunlit growth chambers (Koito Electric Industries, Shizuoka, Japan). Each growth chamber was a cube with 2 m sides. The top, west, south and east sides of each chamber were made of glass, and the four chambers were lined up facing south in a sunlit glasshouse in which air temperature was controlled at 27 °C. The double glasshouse structure alleviated the variation of air temperature within each chamber (Supplementary data Fig. S2). The air temperature of the chambers was set at 15, 20, 25 and 30 °C, and humidity was set at 70 %. During the dark period, the air temperature was controlled at the pre-set temperature, whereas it was raised by up to about 5 °C during the light period depending on the solar radiation (Supplementary data Figs S3 and S4). We calculated the regression equations of the air temperatures in the growth chambers against the outside solar radiation, and used them to estimate the mean air temperatures in the growth chambers during the cultivation period. The estimated mean air temperatures in the growth chambers during the cultivation were 15.4, 21.8, 26.6 and 31.7 °C for the four chambers containing *B. gymnorrhiza*, and 15.2, 21.5, 26.3 and 31.4 °C for those containing *R. stylosa* (Supplementary data Fig. S5). We used the estimated mean air temperature in each growth chamber during the cultivation rather than the pre-set temperature for all data analyses on growth parameters. Standard deviations of air temperature, light intensity and humidity measurements within the chambers were 0.4 °C, 12.8 µmol m^−2^ s^−1^ and 1.6 %, respectively, and standard deviations of light intensity and humidity among the chambers were 13.2 µmol m^−2^ s^−1^ and 1.8 %, respectively (Supplementary data Fig. S3). The light intensity was affected by weather conditions, but neither the light intensity nor the humidity differed significantly among chambers (Supplementary data Table S1). Air temperature, light intensity and humidity did not differ significantly within a chamber (Supplementary data Table S2). The temperatures at leaves and roots were not significantly different (Supplementary data Fig. S6). Plant heights on the initial day of the cultivation were 27.8 ± 2.53 cm (mean ± s.d.) for *B. gymnorrhiza*, and 29.4 ± 2.45 cm for *R. stylosa.* The positions of the pots were rotated within each growth chamber twice a week. The plants grown in the chambers that were set at 15, 20, 25 and 30 °C are referred to as 15 °C-, 20 °C-, 25 °C- and 30 °C-growth plants, respectively. The set growth temperature is referred to as the growth condition (e.g. 15 °C growth condition).

### Measurement of growth rate, biomass allocation and N uptake rate

On the initial day and 56th day of the cultivation, we sampled seedlings of both species to analyse growth. During the 56 d cultivation period, both species were in the juvenile phase, and developed 2–6 leaves; from four to 8–10 leaves in *B. gymnorrhiza* and from four to 6–8 leaves in *R. stylosa*. Neither species flowers during the juvenile phase, which lasts for 6–8 years. No seedlings showed dead leaves during the 56 d cultivation period. The RGR (g g^−1^ d^−1^), net assimilation rate (NAR; g m^−2^ d^−1^) and leaf area ratio (LAR; m^2^ g^−1^) were determined for individual plants at each growth temperature according to the following equations (Lambers *et al.*, 2008).


$$\begin{array}{l} RGR=\frac{1}{W}\frac{dW}{dt}=\frac{\ln W_{f}-\ln W_{i}}{t_{f}-t_{i}} \end{array}$$ (1)

$$\begin{array}{l} NAR=\frac{1}{A}\frac{dW}{dt}=\frac{W_{f}-W_{i}}{A_{f}-A_{i}}\frac{\ln A_{f}-\ln A_{i}}{t_{f}-t_{i}} \end{array}$$ (2)

$$\begin{array}{l} LAR=RGR/NAR \end{array}$$ (3)

where *W*_i_ and *W*_f_ are total dry mass (g), and *A*_i_ and *A*_f_ are leaf area (m^2^) on the initial day (*t*_i_) and the final day of cultivation (*t*_f_), respectively.

Leaf mass ratio (LMR; g g^−1^), root mass ratio (RMR; g g^−1^) and leaf mass per unit leaf area (LMA; g m^−2^) were determined for individual seedlings from the dry weights of the whole plant, all leaves, all roots and the total area of all leaves on the final day of cultivation (Lambers *et al.*, 2008).

When seedlings were individually transplanted into pots, another eight seedlings per species were randomly selected from the tray, and the fresh weights of the whole seedlings and of their leaves, stems and roots were determined. All leaves from each of the eight seedlings were scanned, and their areas were measured by ImageJ software (Abramoff *et al.*, 2004). The leaves, stems and roots were then dried at 80 °C until their weight became constant; this final weight was classed as the dry weight. The mean ratios of their dry weight to fresh weight, and the dry weight of each of their organs were used to estimate the initial dry weight of each organ of the pot seedlings in the cultivation experiment. At the end of the 56 d cultivation period, five seedlings per species in each chamber were randomly selected and divided into leaves, stems and roots, and used to measure the O_2_ respiration rate. The leaf area of each seedling was then measured as above and all organ samples were dried at 80 °C until their weight became constant, and the dry weight was measured.

Subsequently, the samples were ground into fine powder, 10 mg of each sample was wrapped in tin foil, and N and carbon (C) contents were measured with an elemental analyser (Flash EA 1112, Thermo Electron Corp., Minneapolis, MN, USA). Each sample was measured three times and the mean value was determined. The C contents were used as organic C contents (C_org_). The net rate of N uptake per unit root dry mass (NNUR; nmol N g^−1^ s^−1^) was determined for each seedling as follows (White, 1972):


$$\begin{array}{l} NNUR\;=\frac{\ln W_{rootf}-\ln W_{rooti}}{t_{f}-t_{i}}\times \frac{N_{f}-N_{i}}{W_{rootf}-W_{rooti}}\; \end{array}$$ (4)

where *W*_rooti_ and *W*_rootf_ are the root dry mass (g) at the initial day (*t*_i_) and final day (*t*_f_), respectively; and *N*_i_ and *N*_f_ are the total N amounts in the plant (nmol) at *t*_i_ and *t*_f_, respectively.

### Measurement of oxygen respiration rates

At the end of the 56 d cultivation period, O_2_ respiration rates of leaves and roots (R; nmol O_2_ g d. wt^−1^ s^−1^) were measured individually in five randomly selected seedlings per species as follows. Leaves and roots were detached into separate 50 mL glass vials, and O_2_ consumption rates were measured with a fluorescence O_2_ sensor (FDO 925, Xylem Analytics, Weilheim, Bavaria, Germany). During measurement, the samples were kept at 15, 20, 25 or 30 °C to match their growth temperature. Measurements were conducted in a gaseous phase with all leaves of one seedling, and in a liquid phase with about half of the root system of one seedling. For root measurements, each vial was filled with air-saturated nutrient solution (1:2000-diluted Hyponex) containing 50 mm MES buffer (pH 6.5). A constant rate of O_2_ uptake was recorded at the corresponding temperature for 15 min. Each sample was retrieved from its vial, dried at 80 °C until the weight became constant and then weighed to give the dry weight.

The O_2_ respiration rates for the growth of leaves and roots (R_g_; nmol O_2_ g d. wt^−1^ s^−1^) were obtained by multiplying RGR by the regression coefficient for growth (g; mol C g d. wt^−1^) of each organ, assuming that the respiratory quotient RQ = 1 (Hachiya and Noguchi, 2008). The RQ has been reported to be around 1 in *Bruguiera* and *Rhizophora* species (Chapman, 1962; Brown *et al.*, 1969), and in many other plants (Lambers and Oliveira, 2019). RGR and *g* at 15, 20, 25 and 30 °C were calculated by using fitted curves for measured RGR or *g* vs. the estimated mean growth temperature. O_2_ respiration rate for leaf maintenance (R_m_; nmol O_2_ g d. wt^−1^ s^−1^) and O_2_ respiration rate for root maintenance and N uptake (R_m_ + R_u_; nmol O_2_ g d. wt^−1^ s^−1^, where R_u_ is the root O_2_ respiration rate for N uptake) were obtained by subtracting R_g_ from the O_2_ respiration rate for each organ. R_u_ (nmol O_2_ g d. wt^−1^ s^−1^) was obtained by multiplying the specific cost of nitrate uptake (*c*_u_; mol O_2_ mol^−1^ NO_3_^−1^) by NNUR and the ratio of nitrate N to total N in the nutrient solution (mol mol^–1^). The ratio was set to 0.75 because the estimated concentration of nitrate N was 2.34 mmol L^–1^ and that of total N was 3.13 mmol L^–1^ in 1:2000-diluted Hyponex solution. NNUR values at 15, 20, 25 and 30 °C were calculated by using fitted curves of the measured NNUR vs. the estimated mean growth temperature. Theoretical R_u_ can be obtained under several assumptions from the following equations (Bouma *et al.*, 1996; Scheurwater *et al.*, 1999; Kurimoto *et al.*, 2004*a*).


$$\begin{array}{l} R_{u}=\frac{I}{I-E}\times c_{u}\times NNUR\times a \end{array}$$ (5)

$$\begin{array}{l} c_{u}=\frac{\frac{H^{+}}{I_{j}}\times M_{j}}{\frac{H^{+}}{P}\times \frac{P}{O_{2}}}=\frac{2}{\frac{P}{O_{2}}} \end{array}$$ (6)

where *I* is the influx of nitrate into the cytoplasm, *E* is the efflux (or leak) of nitrate out of the cytoplasm (Scheurwater *et al.*, 1999), *a* is the proportion of nitrate N in the total N in the nutrient solution (mol mol^−1^), H^+^/*I*_*j*_ is the number of protons required for a membrane symport of nitrate (2 mol H^+^ mol^−1^ NO_3_^−^; Crawford and Glass, 1998), *M*_*j*_ is the number of membranes to be crossed actively (= 1; the plasma membrane), H^+^/P is the amount of protons pumped across the plasma membrane by the H^+^-ATPase per hydrolysis of one ATP to ADP (= 1 mol H^+^ mol^−1^ ATP; Crawford and Glass, 1998), and P/O_2_ is the efficiency of oxidative phosphorylation [29/6 when only the cytochrome pathway (CP) is engaged, and 11/6 when only the alternative pathway (AP) is engaged; Amthor, 1994)]. Therefore, (1) efflux of nitrate and (2) electron partitioning to AP (*τ*_a_) have positive effects on R_u_. For example, when no efflux of nitrate occurs (*E* = 0) and all electrons flow to the CP (P/O_2_ = 29/6), $\frac{I}{I-E}\times c_{u}$ will be 0.41, and when nitrate efflux is 60 % (*E* = 0.6) and electrons flow equally to the CP and AP (P/O_2_ = 20/6), $\frac{I}{I-E}\times c_{u}$will be 1.50. We estimated the R_u_ in the theoretical range of $\frac{I}{I-E}\times c_{u}$ values as being 0.41–1.50 mol O_2_ mol^–1^ NO_3_^–^.

### Determination of protein contents of leaves and roots

At the end of the cultivation experiment, the leaves and roots of each seedling were collected and approx. 200 mg per seedling was stored at −80 °C until protein extraction. Each sample was ground with a mortar and pestle in buffer containing 2 % (w/v) SDS 62.5 mm tris(hydroxymethyl)aminomethane-HCl (pH 6.8), 50 mm dithiothreitol, 7.5 % (v/v) glycerol, 0.01 % (w/v) bromophenol blue and a protease inhibitor tablet (Roche Diagnostics, Mannheim, Germany). The homogenate was heated at 100 °C for 5 min and centrifuged at 15 000 *g* for 10 min. Protein content of the supernatant was measured according to the method of Peterson (1977) with bovine serum albumin as the standard.

### Estimation of construction costs and regression coefficients for growth of leaves and roots

The construction cost (CC; the amount of glucose required to produce 1 g of biomass, g glucose g^−1^) of leaves and roots was determined as follows (Poorter, 1994; Hachiya and Noguchi, 2008):


$$\begin{array}{l} CC=\left(-1.041+5.077\times C_{org}\right)\times \left(1-m\right)+5.325\times N_{org} \end{array}$$ (7)

where *m* is mineral content, N_org_ is organic N content (g g d. wt^−1^), and C_org_ is organic C content (g g d. wt^−1^).

Mineral content was obtained by multiplying ash content (g ash weight g d. wt^−1^) by 0.67. This coefficient has been used for many plant species (e.g. Vertregt and Penning de Vries, 1987; Hachiya and Noguchi, 2008). Dried samples were combusted at 550 °C for 5 h and the ash was weighed. Organic N content was obtained by subtracting nitrate content from total N content; nitrate content of dried samples was obtained by the colorimetric method (Cataldo *et al.*, 1975) as follows. Each dried sample (10 mg) was extracted with 1 mL of distilled water at 100 °C for 30 min; 0.2 mL of the extract was pipetted into a 50 mL flask and mixed with 0.8 mL of 5 % (w/v) salicylic acid that was dissolved in concentrated H_2_SO_4_. After 20 min at room temperature, 19 mL of 2 n NaOH was added, and the mixture was cooled to room temperature for 10 min. Absorbance at 410 nm was measured with a spectrophotometer (U-1100, Hitachi High-Tech Corporation, Tokyo, Japan).

The regression coefficient for growth (g; mol C g d. wt^−1^) of leaves and roots was determined as follows (Hachiya and Noguchi, 2008):


$$\begin{array}{l} g=\left(CC\times \frac{6}{180.15}\right)-\left(\frac{C_{org}}{12.01}\right) \end{array}$$ (8)

where 180.15 is the molecular weight of glucose (g mol^−1^), 12.01 is the atomic weight of C (g mol^−1^) and 6 is the number of C atoms in one glucose.

### Statistical analysis

Growth temperature dependencies of the parameters, and LMA dependencies of leaf CC were fitted by second-order polynomial regression with a 95 % confidence interval. Linear regression was used to examine relationships between RGR and NAR and between RGR and LAR. For comparison of growth, biomass allocation and morphology among species and set growth conditions, two-way analysis of variance (ANOVA) was conducted after the normality of the raw data was confirmed by a normality test. For comparison of O_2_ respiration rates, construction costs and regression coefficients for growth and protein contents among species, organs and assigned growth temperatures, a multifactor ANOVA was used after normality of the raw data was confirmed as above. Treatments were compared by conducting a Tukey’s multiple-comparison test using the ‘glht’ function in the ‘multcomp’ package (Hothorn *et al.*, 2008) of R v.3.6.2 software (R Core Team, 2017). A type I error rate (*P*) < 0.05 was considered significant.

## RESULTS

### Dependencies of growth parameters on growth temperatures in two mangrove species

**Fig. 1.**
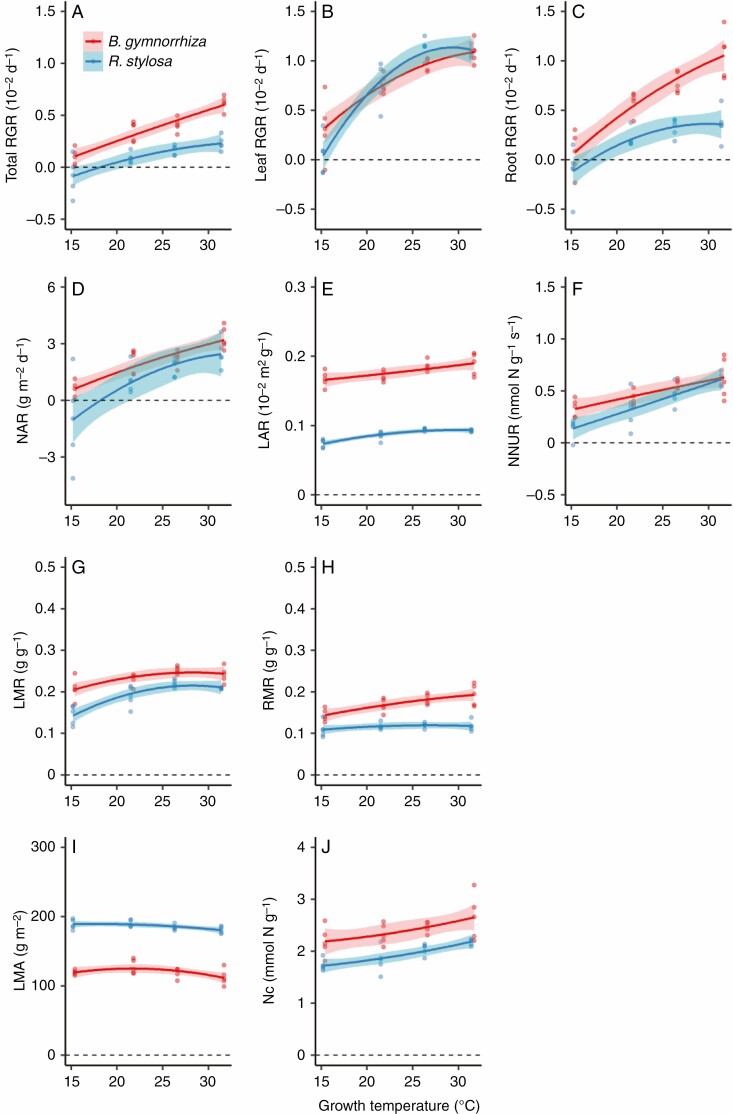
Growth parameters of *Bruguiera gymnorrhiza* and *Rhizophora stylosa* at different growth temperatures. (A) Total relative growth rate (RGR), (B) leaf RGR, (C) root RGR, (D) net assimilation rate, (E) leaf area ratio, (F) net rate of nitrogen uptake per unit root dry mass, (G) leaf mass ratio, (H) root mass ratio, (I) leaf mass per unit leaf area and (J) plant nitrogen content at different growth temperatures. The growth temperatures on the *x*-axis are the estimated mean air temperatures in the growth chambers during the cultivation experiment. Solid lines are fitted curves of the second-order polynomial regression model, and the red and blue shading indicates 95 % confidence intervals. Dashed lines indicate zero on the *y*-axis. Statistical results and the coefficients and intercepts of the regression model are summarized in Supplementry data Tables S3–S5.

**Fig. 2.**
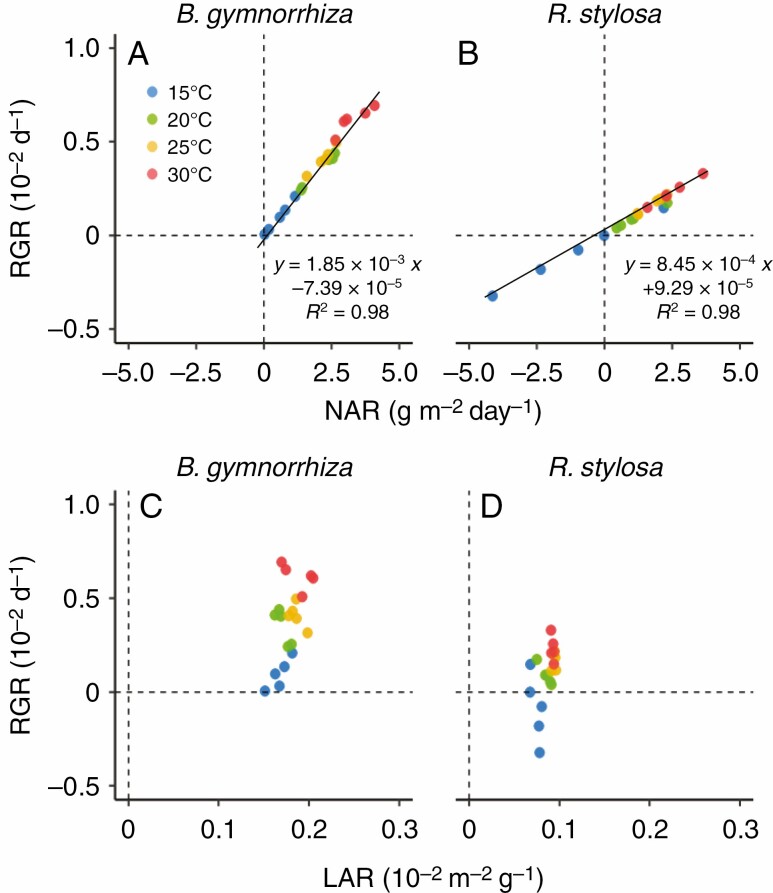
Relationships between (A, B) total RGR and NAR and (C, D) total RGR and LAR in (A, C) *B. gymnorrhiza* and (B, D) *R. stylosa*. Solid lines indicate the fitted linear regression model.

We examined plant growth at four different temperatures (15, 20, 25 and 30 °C) for five plants of each species. On the initial day of the cultivation, the dry weight of the whole plant (mean ± SD) was 9.02 ± 1.42 g for *B. gymnorrhiza* and 13.69 ± 1.39 g for *R. stylosa*. On the final day of the 56 d cultivation period, the mean whole dry weight was 9.61 g (15 °C), 10.53 g (20 °C), 12.13 g (25 °C) and 12.88 g (30 °C) for *B. gymnorrhiza* and 12.60 g (15 °C), 14.87 g (20 °C), 15.29 g (25 °C) and 15.08 g (30 °C) for *R. stylosa.* The RGR of the whole plant (total RGR) differed significantly between the species and among growth temperatures (Fig. 1A; both *P* < 0.001, ANOVA in Supplementary data Table S3). Total RGR was significantly higher in *B. gymnorrhiza* than in *R. stylosa* at 20–30 °C (*P* < 0.05, Tukey’s multiple comparison test in Supplementary data Table S4), and it increased with growth temperature in both species (Supplementary data Table S4, S5). In the ANOVA, the interaction term between species and growth temperature was significant, indicating that the temperature dependencies of total RGR differed between the two species (*P* < 0.05, Supplementary data Table S3); the increment of total RGR against growth temperature was higher in *B. gymnorrhiza* than in *R. stylosa*. Total RGR is determined by NAR and LAR (RGR = NAR × LAR). NAR differed significantly between the species (Fig. 1D; *P* < 0.01, Supplementary data Table S3) and among growth temperatures (*P* < 0.001, Supplementary data Table S3). There was no significant interaction between the effects of species and growth temperature on NAR (Supplementary data Table S3). Despite the significant result by ANOVA, the interspecific differences were not significant at any growth temperature in the Tukey’s multiple comparison test (Supplementary data Table S4). Values of total RGR and NAR were negative for four out of five *R. stylosa* plants under the 15 °C growth condition. Total RGR and NAR were strongly correlated with each other in both species (both *R*^2^ = 0.98), but the regression coefficient in *B. gymnorrhiza* was 2.19 times that in *R. stylosa* (Fig. 2A, B). The LAR differed significantly between the species and among growth temperatures (Fig. 1E; *P* < 0.001, Supplementary data Table S3). There was no significant interaction between the effects of species and growth temperature on LAR (Supplementary data Table S3). Although LAR significantly increased with growth temperature, the temperature dependencies of LAR were small in both species (Fig. 1E; Supplementary data Table S5).

The above results suggest that the differences in total RGR among growth temperatures can be explained by those in NAR in both species. In *R. stylosa*, the negative values of total RGR of 15 °C-growth plants may be due to the negative values of NAR, which suggests that photosynthesis and/or respiration are not well regulated in *R. stylosa* grown under this low temperature. The fitted curves for measured total RGR vs. estimated mean growth temperature indicated that total RGR became zero at 12.2 °C in *B. gymnorrhiza* and at 18.1 °C in *R. stylosa* (Table 1; Supplementary data Table S5).

**Table 1.** Growth temperatures at which fitted curves of RGR become zero in whole plants, leaves and roots of *B. gymnorrhiza* and *R. stylosa*

	B. gymnorrhiza	R. stylosa
	Temperature (°C)	
Whole plant	12.2	18.1
Leaf	11.8	14.9
Root	14.5	17.1

The interspecific difference in total RGR could be explained mainly by LAR being significantly higher in *B. gymnorrhiza* than in *R. stylosa* (Fig. 1E; *P* < 0.001, Supplementary data Tables S3 and S4). LAR is determined by specific leaf area, SLA, and LMR (LAR = SLA × LMR). We compared LMR and LMA, the reciprocal of SLA. LMR differed significantly between the species and among growth temperatures (Fig. 1G; *P* < 0.001, Supplementary data Table S3). There was no significant interaction between the effects of species and growth temperature on LMR (Supplementary data Table S3). LMR was higher in *B. gymnorrhiza* than in *R. stylosa* under 15 and 20 °C growth conditions (Supplementary data Table S4). In both species, LMR increased significantly as growth temperature increased from 15 to 25 °C, and was saturated under the 25 and 30 °C growth conditions (Fig. 1G; Supplementary data Tables S4 and S5).

The LMA differed significantly between the species (Fig. 1I; *P* < 0.001, Supplementary data Table S3) and among growth temperatures (*P* < 0.05, Supplementary data Table S3). There was no significant interaction between the effects of species and growth temperature on LMA. Although LMA slightly decreased with growth temperature in both species, the decrease was small (Fig. 1I; Supplementary data Table S5) and was not significant by Tukey’s multiple comparison test (Supplementary data Table S4). LMA was significantly higher in *R. stylosa* than in *B. gymnorrhiza* under all growth conditions, probably because *R. stylosa* has thicker leaves than *B. gymnorrhiza* (Fig. 1I; Supplementary data Table S4); the extent of interspecific difference in LMA was higher than that in LMR.

Regarding N acquisition, RMR differed significantly between the species and among the growth temperatures (Fig. 1H; *P* < 0.001, Supplementary data Table S3). The interaction between species and growth temperature was significant, indicating that the temperature dependencies of RMR differed between the two species (*P* < 0.01, Supplementary data Table S3). In the 15–25 °C growth conditions, RMR was significantly larger in *B. gymnorrhiza* than in *R. stylosa*; RMR increased with growth temperature in *B. gymnorrhiza*, but was almost constant in *R. stylosa* (Supplementary data Tables S4 and S5). The NNUR and N content of the plant (Nc) differed significantly between the species (Fig. 1F, J; *P* < 0.01 for NNUR and *P* < 0.001 for Nc, Supplementary data Table S3) and among growth temperatures (*P* < 0.001 for both NNUR and Nc, Supplementary data Table S3). The NNUR and Nc increased with growth temperature in both species; there was no significant interaction between the effects of species and growth temperature on either parameter (Supplementary data Tables S3 and S5). The interspecies difference was only significant for Nc under the 20 and 30 °C growth conditions in the Tukey’s multiple comparison test (Supplementary data Table S4). *Rhizophora stylosa* had fewer roots and thus slower rates of N uptake than *B. gymnorrhiza*, especially at the low temperature.

Leaf RGR differed significantly among growth temperatures (Fig. 1B; *P* < 0.001, Supplementary data Table S3), but not between species. There was no significant interaction between the effects of species and growth temperature on leaf RGR (Supplementary data Table S3). The fitted curves for leaf RGR vs. estimated mean growth temperature peaked at 34.5 °C in *B. gymnorrhiza* and at 29.3 °C in *R. stylosa*; the peak was estimated to be where the primary differential coefficient (slope) of the fitted curve became zero (Supplementary data Table S5). In these fitted curves, RGR became zero at 11.8 °C in *B. gymnorrhiza* and at 14.9 °C in *R. stylosa* (Table 1). Unlike leaf RGR, root RGR differed significantly between the species as well as among growth temperatures (Fig. 1C; both *P* < 0.001, Supplementary data Table S3). Furthermore, for root RGR, the interaction between species and growth temperature was significant, indicating that the temperature dependencies of root RGR differed between the two species (*P* < 0.01, Supplementary data Table S3). Root RGR increased with growth temperatures across the entire range in *B. gymnorrhiza*, but was saturated when the plants were cultured under 25 or 30 °C growth conditions in *R. stylosa*. Root RGR was significantly higher in *B. gymnorrhiza* than in *R. stylosa* when the plants were cultured under ≥20 °C growth conditions (Supplementary data Table S4). In the fitted curves for root RGR vs. estimated mean growth temperature (Supplementary data Table S5), root RGR became zero at 14.5 °C in *B. gymnorrhiza* and at 17.1 °C in *R. stylosa* (Table 1).

### Construction cost and protein content of leaves and roots

The above results indicate that biomass allocation to leaves and roots (i.e. LMR and RMR, respectively) was larger in *B. gymnorrhiza* than in *R. stylosa*. We therefore determined the chemical compositions and estimated the construction costs of leaves and roots in both species.

**Fig. 3.**
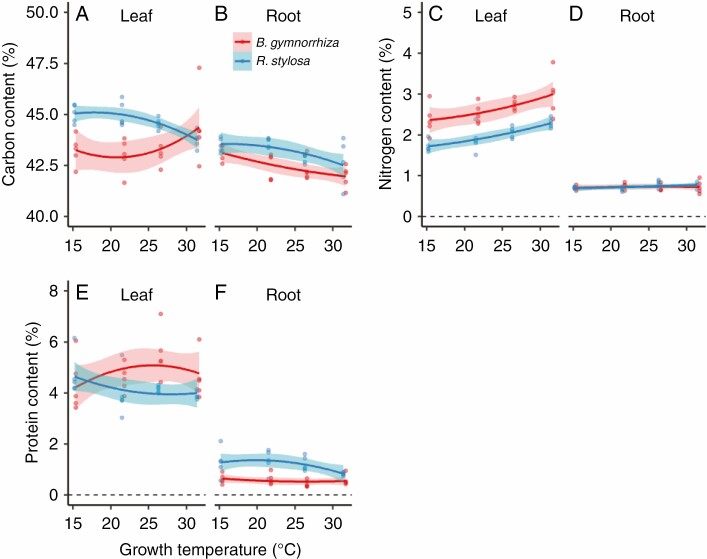
Contents of (A, B) carbon, (C, D) nitrogen and (E, F) protein in (A, C, E) leaves and (B, D, F) roots of *B. gymnorrhiza* and *R. stylosa*. For other details, see the legend of Fig. 1.

The C content differed significantly between the species (Fig. 3A, B; *P* < 0.001, ANOVA), between organs (*P* < 0.001, ANOVA) and among growth temperatures (*P* < 0.01, ANOVA) (Supplementary data Table S3). The ANOVA results showed a significant three-way interaction among species, organs and growth temperature, indicating that the temperature dependencies of C content differed among the combinations of species and organs (*P* < 0.01, Supplementary data Table S3). Leaf C content was significantly lower in *B. gymnorrhiza* than in *R. stylosa* at 15 and 20 °C growth temperatures (Fig. 3A; Supplementary data Table S4), but root C content did not differ significantly between the species (Fig. 3B; Supplementary data Table S4). The fitted curves for C content vs. estimated mean growth temperature showed slightly different trends between the leaves of the two species: C content increased with growth temperature at ≥20 °C in *B. gymnorrhiza* leaves, but slightly decreased with growth temperature in *R. stylosa* leaves. However, C content decreased with growth temperature across the whole temperature range tested in the roots of both species (Fig. 3A, B; Supplementary data Table S5).

The N content differed significantly between the species, between organs and among growth temperatures (Fig. 3C, D; *P* < 0.001, Supplementary data Table S3). Leaf N content was higher in *B. gymnorrhiza* than in *R. stylosa* (Fig. 3C; *P* < 0.001, Supplementary data Tables S3 and S4), and was higher in leaves than in roots in both species (Fig. 3C, D; *P* < 0.001, Supplementary data Tables S3 and S4). The interaction between the effects of species and organs on N content was significant, indicating that the difference in N content between leaves and roots differed between the two species (*P* < 0.001, Supplementary data Table S3): this difference was larger in *B. gymnorrhiza* than in *R. stylosa*. There was also a significant interaction between the effects of organ and growth temperature on N content, indicating that the temperature dependencies of N content differed between organs (*P* < 0.001, Supplementary data Table S3). In both species, the N content increased with growth temperature in the leaves (Fig. 3C; Supplementary data Table S4), but remained constant in the roots (Fig. 3D; Supplementary data Table S4).

Because most of the N taken up by the plant is used for protein synthesis, we examined the protein contents of leaves and roots. Protein content was higher in the leaves than in the roots in both species (Fig. 3E, F; *P* < 0.001, Supplementary data Tables S3 and S4). There was a significant interaction between species and organ (*P* < 0.001, Supplementary data Table S3), indicating that the difference in protein content between leaves and roots differed between the two species; this difference was higher in *B. gymnorrhiza* than in *R. stylosa* (Fig. 3E, F). The interaction between species and growth temperature was also significant (*P* < 0.05, Supplementary data Table S3), indicating that the temperature dependencies of protein content differed between the two species. The fitted curves for protein content vs. estimated mean growth temperature showed that protein content tended to increase with growth temperature in the leaves of *B. gymnorrhiza* but to decrease with growth temperature in those of *R. stylosa* (Fig. 3E; Supplementary data Table S5).

Nitrate N content differed between the species (Supplementary data Fig. S7A, B; *P* < 0.01, Supplementary data Table S3), between organs (*P* < 0.01, Supplementary data Table S3) and among growth temperatures (*P* < 0.001, Supplementary data Table S3). The interaction between organs and growth temperature was significant (*P* < 0.05, Supplementary data Table S3), indicating that the temperature dependencies of nitrate N content differed between leaves and roots. Nitrate N content decreased with growth temperature in leaves, but was almost constant in roots (Supplementary data Fig. S7A, B).

Mineral content differed significantly among growth temperatures (Supplementary data Fig. S7C, D; *P* < 0.001, Supplementary data Table S3). The three-way interaction among species, organs and growth temperature was significant (*P* < 0.05, Supplementary data Table S3), indicating that the temperature dependencies of mineral content differed among the combinations of species and organs. Leaf mineral content decreased with growth temperature in *B. gymnorrhiza*, but increased with growth temperature in *R. stylosa* (Supplementary data Fig. S7C, D). Root mineral content was almost constant in the two species.

**Fig. 4.**
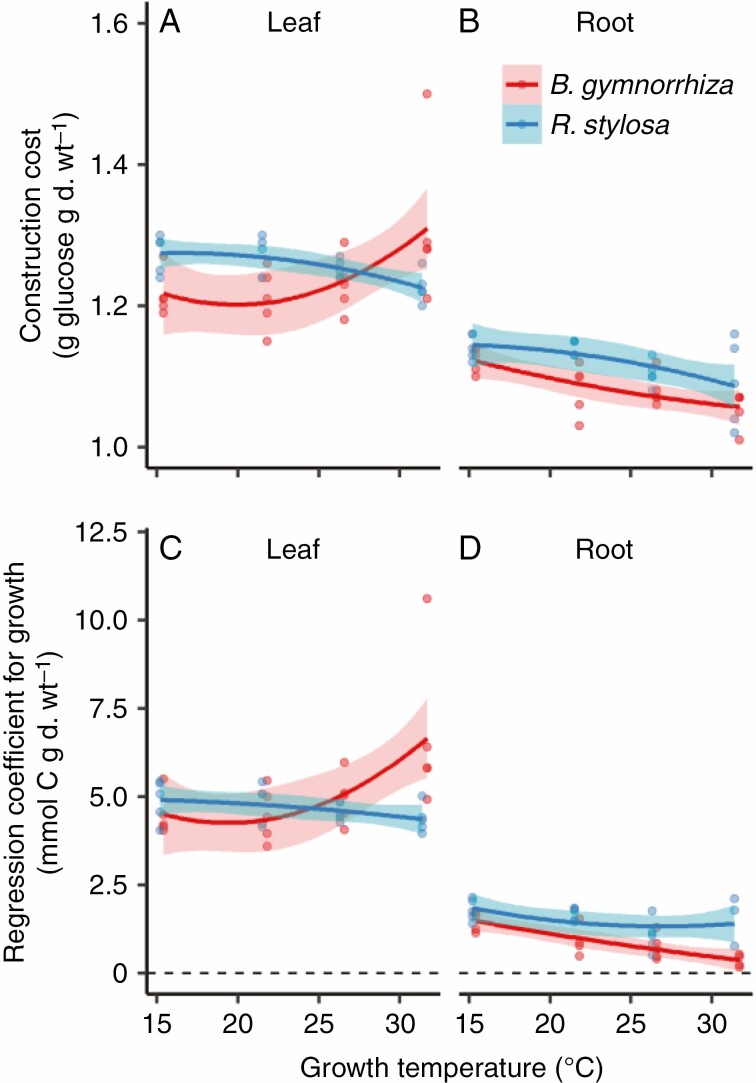
Construction cost and regression coefficient for growth of (A, C) leaves and (B, D) roots in *B. gymnorrhiza* and *R. stylosa*. For other details, see the legend of Fig. 1.

**Fig. 5.**
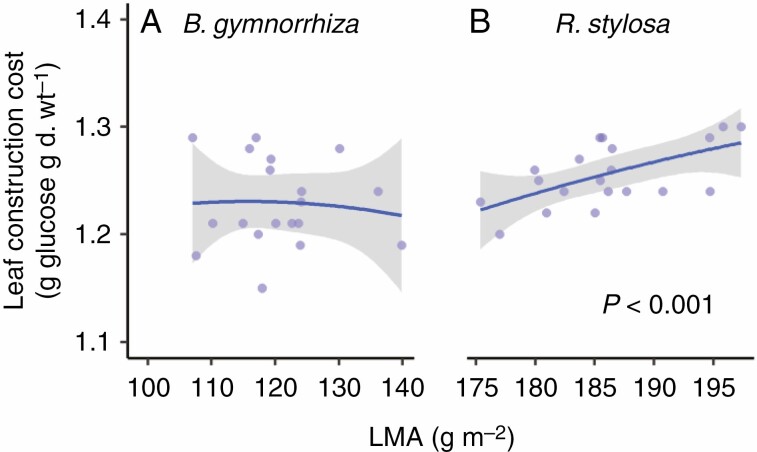
Relationships between construction cost and leaf mass per unit leaf area of (A) *B. gymnorrhiza* and (B) *R. stylosa*. Solid lines indicate fitted curves of the second-order polynomial regression model, and grey shading indicates the 95 % confidence intervals.

Using the data on N, C, nitrate and mineral contents and eqn (7), we estimated the CC of leaves and roots. The CC differed significantly between the species (Fig. 4A, B; *P* < 0.05, Supplementary data Table S3) and between organs (*P* < 0.001, Supplementary data Table S3). CC was higher in the leaves than in the roots in both species (Fig. 4A, B; *P* < 0.001, Supplementary data Tables S3 and S4). There was a significant three-way interaction among species, organs and growth temperature (*P* < 0.01, Supplementary data Table S3), indicating that temperature dependencies of CC differed among the combinations of species and organs. The fitted curves of CC vs. estimated mean growth temperature showed that CC increased with growth temperature in the leaves of *B. gymnorrhiza*, but slightly decreased with growth temperature in the leaves of *R. stylosa* and in the roots of both species (Fig. 4A, B; Supplementary data Table S5). In *R. stylosa*, a positive relationship between leaf CC and LMA was observed (*P* < 0.001, Fig. 5).

The regression coefficient for growth, g, calculated using the CC data and eqn (8), differed significantly between organs (*P* < 0.001, Supplementary data Table S3); it was significantly higher in the leaves than in the roots in both species (Fig. 4C, D; Supplementary data Table S4). The three-way interaction among species, organ and growth temperature was significant (*P* < 0.001, Supplementary data Table S3), indicating that the temperature dependencies of g differed among the combinations of species and organs. As observed for CC, the fitted curves of *g* vs. estimated mean growth temperature showed that *g* increased with growth temperature in the leaves of *B. gymnorrhiza*, but slightly decreased in the leaves of *R. stylosa* and the roots of both species (Fig. 4C, D; Supplementary data Table S5).

### Leaf and root O_2_ respiration rates at different growth temperatures

**Fig. 6.**
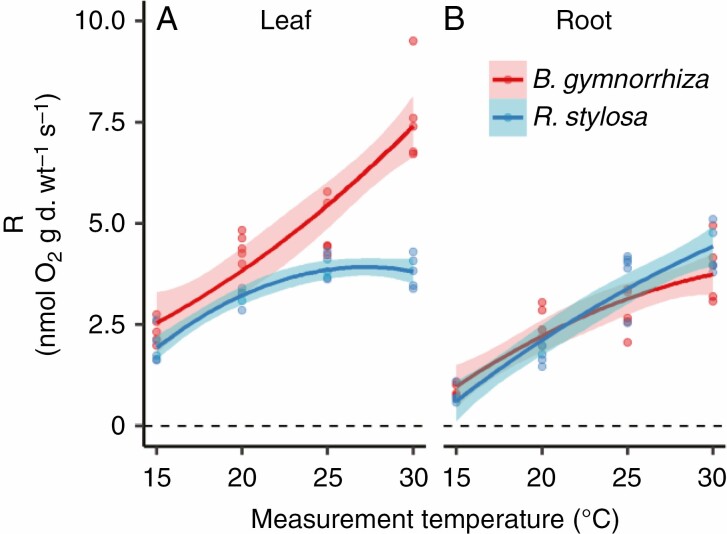
O_2_ respiration rates (R) of (A) leaves and (B) roots of *B. gymnorrhiza* and *R. stylosa*. For other details, see the legend of Fig. 1.

Since we observed differences in growth parameters and their dependencies on growth temperature, we examined leaf and root R, which are related to ATP production for growth, maintenance and N uptake. R significantly differed between species, between organs and among growth temperatures (Fig. 6A, B; *P* < 0.001, Supplementary data Table S3). We observed a significant three-way interaction among species, organs and growth temperature (*P* < 0.001, Supplementary data Table S3), indicating that the temperature dependencies of R differed among the combination of species and organs. Leaf R increased steadily with growth temperature in *B. gymnorrhiza*, but peaked at 25 °C in *R. stylosa* (Fig. 6A; Supplementary data Table S4). The fitted curve of leaf R vs. estimated mean growth temperature (Supplementary data Table S5) peaked at 27.2 °C in *R. stylosa*. The temperature dependencies of leaf R were larger in *B. gymnorrhiza* than in *R. stylosa*, and leaf R in *B. gymnorrhiza* at 30 °C was twice that in *R. stylosa*. In contrast, root R was similar in both species, and increased significantly with growth temperature (Fig. 6B; Supplementary data Table S4). The R values at the temperatures where RGR became zero were 1.89 and 1.91 nmol O_2_ g d. wt^−1^ s^−1^ in the leaves of *B. gymnorrhiza* and *R. stylosa*, respectively, and 0.84 and 1.28 nmol O_2_ g d. wt^−1^ s^−1^ in the roots of *B. gymnorrhiza* and *R. stylosa*.

**Fig. 7.**
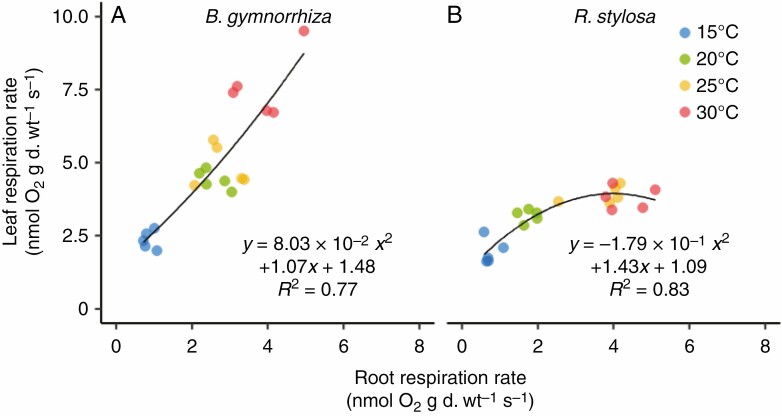
Relationships between leaf and root O_2_ respiration rates of (A) *B. gymnorrhiza* and (B) *R. stylosa*. Solid lines indicate fitted curves of the second-order polynomial regression model.

Positive correlations were observed between leaf R and root R in both species (Fig. 7), but the relationship differed between the two species. In *B. gymnorrhiza*, both leaf R and root R increased with growth temperature, but in *R. stylosa* leaf R did not increase from 25 to 30 °C, whereas root R did increase (Fig. 6A, B).

### O_2_ respiration costs for growth, maintenance and N uptake

**Fig. 8.**
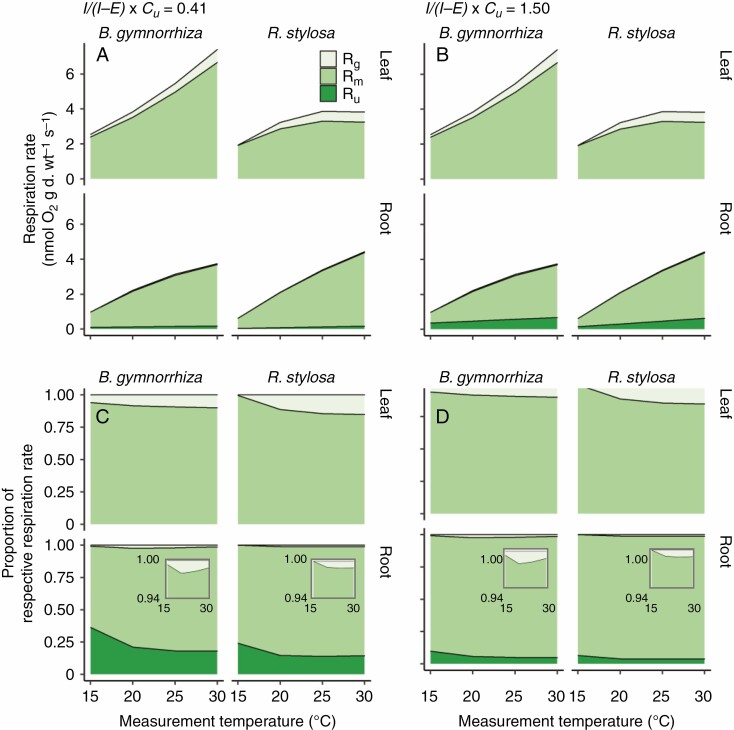
O_2_ respiration rates for growth (R_g_), maintenance (R_m_) and nitrogen uptake (R_u_) at different growth temperatures in *B. gymnorrhiza* and *R. stylosa*. In the R_u_ calculation, $\frac{I}{I-E}\times c_{u}$ was assumed to be 0.41 (A, C) or 1.50 (B, D). *I*, influx of nitrate into the cytoplasm; *E*, efflux (or leak) of nitrate out of the cytoplasm; *c*_u_, specific cost of nitrate uptake. The upper parts show absolute values (A, B), and the lower parts show the proportions of R_g_, R_m_ and R_u_ in total respiration rates (C, D). Enlarged graphs for roots are shown in (C) and (D). The temperatures on the *x*-axis are the measurement temperatures of O_2_ respiration rates.

Using the data for g, RGR and R, we calculated respiration rates for growth (R_g_) and estimated respiration rates for maintenance (R_m_) and N uptake (R_u_) in roots (Fig. 8). RGR and *g* at 15, 20, 25 and 30 °C were calculated from the fitted curves of measured RGR or *g* vs. the estimated mean growth temperature (Figs 1 and 4). In both species, R_g_ was low, especially in roots (Fig. 8A, B), and thus leaf R_m_ and root R_m_ accounted for most of R (Fig. 8C, D). The contribution of R_g_ to R was <20 % in leaves and <5 % in roots in both species (Fig. 8C, D).

Both leaf R_m_ and leaf R_g_ increased steadily with growth temperature in *B. gymnorrhiza*, but were saturated at 25 and 30 °C in *R. stylosa* (Fig. 8A, B). Although both leaf R_m_ and leaf R_g_ responded positively to growth temperature, their temperature dependencies differed from each other. Thus, the proportion of R_g_ in R varied among growth temperatures in both species (Fig. 8C, D). This proportion in leaves increased with growth temperature and was saturated at 25 and 30 °C in *B. gymnorrhiza* and at 20–30 °C in *R. stylosa*, and was higher in *R. stylosa* than in *B. gymnorrhiza* at temperatures of ≥20 °C (Fig. 8C, D). Root R_g_ was small in both species (Fig. 8).

We estimated R_u_ using the NNUR data, the assumed *c*_u_ values and the ratio of nitrate N to total N in the nutrient solution. R_u_ increased with growth temperature (Fig. 8A, B) owing to the increase in NNUR with growth temperature. The NNUR data at 15, 20, 25 and 30 °C were calculated from the fitted curves of measured NNUR vs. the estimated mean growth temperature (Fig. 1F). Irrespective of the assumed values ($\frac{I}{I-E}\times c_{u}$ = 0.41; *E* = 0 and P/O_2_ = 29/6 in Fig. 8A, or $\frac{I}{I-E}\times c_{u}$= 1.50; *E* = 0.6 and P/O_2_ = 20/6 in Fig. 8B), the proportion of R_u_ in R was 0.1–0.3 and decreased with growth temperature (Fig. 8C, D).

## DISCUSSION

### Comparison of growth parameters in two mangrove species

In both mangrove species used in this study, growth temperature affected physiological parameters related to growth (NAR and NNUR) rather than structural parameters (LAR and RMR) (Fig. 1). These results are consistent with the reported data on evergreen perennials (Tjoelker *et al.*, 1999; Bruhn *et al.*, 2007). The two species differed in some growth parameters and in their responses to growth temperature. At the same age, *B. gymnorrhiza* had higher leaf and root mass than *R. stylosa* (Fig. 1G, H) and invested more photosynthates into roots at high growth temperature, resulting in higher RGR than in *R. stylosa*.

We also detected a structural difference between the leaves of these species. *Rhizophora stylosa* had higher LMA (i.e. thicker leaves) than *B. gymnorrhiza* (Fig. 1I). This is consistent with the results of Saenger and West (2016), in which SLA of mature trees was higher in *B. gymnorrhiza* than in *R. stylosa* at 13 locations along the Australian coast. A positive relationship between LMA and CC has been reported in deciduous trees due to thick leaves, which have costly cellular components such as lignin (Niinemets, 1999). We found that leaf CC was positively related to LMA in *R. stylosa*, whereas there was no relationship in *B. gymnorrhiza* (Fig. 5). This suggests that a C cost is required to produce the thick leaves of *R. stylosa*. Despite the differences in leaf and root CC between the two mangrove species, the measured leaf and root CCs of both species were within the reported range of temperate terrestrial plants, 1.2–1.9 g glucose g^−1^ (Niinemets, 1999; Martίnez *et al.*, 2002a, b; van Iersel, 2006; Laureano *et al*., 2008, 2013). Therefore, the leaf respiratory features of these two mangrove species are no more costly than those of terrestrial plants.

Plant mass at the end of the 56 d cultivation experiment was higher at higher growth temperatures. Regardless of changes in growth environments such as growth temperature, the RGR is known to decrease as plant mass increases because of ontogenetic drift; the decline in the RGR of tree seedlings with size is concomitant with a decline in the LMR and an increase in the stem mass fraction (Walters *et al.*, 1993; Cornelissen *et al.*, 1996). Although RGR and LMR were rather higher at the higher growth temperatures in which plant mass was higher, we cannot exclude the possibility that RGR at these temperatures was underestimated in our study. If there was ontogenetic drift between the growth temperatures, the difference between low and high growth temperatures may be larger than we observed. In our study, the difference in mean whole dry weight among growth temperatures at the 56th day of cultivation was 3.27 g for *B. gymnorrhiza* and 2.69 g for *R. stylosa*. By using the data for Betulaceae species in Walters *et al.* (1993), we estimate that a 3.5 g plant mass difference may correspond to a difference in RGR of about 0.005 d^−1^. Betulaceae species with higher RGR tend to have higher ontogenetic drift than those with lower RGR (Walters *et al.*, 1993). The RGRs of Betulaceae species in Walters *et al.* (1993) are about ten times that of the two Rhizophoraceae species in our study, and thus the ontogenetic drift of the Rhizophoraceae species may be smaller than the estimated value.

### Different responses of two mangrove species to limiting growth temperatures

Our finding that total, leaf and root RGR reached zero at higher temperatures in *R. stylosa* than in *B. gymnorrhiza* in the fitted curves of RGR vs. estimated mean growth temperature suggests that the seedlings of *R. stylosa* are more sensitive to low temperatures than those of *B. gymnorrhiza*. Our results also suggest that the roots of both species are more sensitive to low temperature than are the leaves, since both species had difficulty in growing roots at below threshold temperatures (i.e. temperature at which RGR reached zero). The O_2_ respiration rates of the two mangrove species at the threshold temperatures (1.89 and 1.91 nmol O_2_ g d. wt^−1^ s^−1^ in leaves of *B. gymnorrhiza* and *R. stylosa*, respectively, and 0.84 and 1.28 nmol O_2_ g d. wt^−1^ s^−1^ in roots of *B. gymnorrhiza* and *R. stylosa*) were not higher than O_2_ respiration rates of temperate terrestrial plants (Zha *et al.*, 2001; van Iersel, 2006; Laureano *et al*., 2008, 2013; Atkin *et al.*, 2015; Hernández-Montes *et al.*, 2019), suggesting that the distribution of the two mangroves in warm areas may not be caused by their high requirement for respiratory ATP at low growth temperatures. In the current study, at 15 °C growth temperature, the rates of N uptake were lower in *R. stylosa* roots than in *B. gymnorrhiza* roots; this may be a reason for the lower growth rate of *R. stylosa* and its higher sensitivity to low temperature.

The leaf O_2_ respiration rate of *B. gymnorrhiza* tended to increase steadily with growth temperature (15–30 °C), whereas it peaked at 27.2 °C in *R. stylosa*. We have previously reported that rates of leaf CO_2_ respiration and leaf assimilation of *R. stylosa* are saturated at 25 °C growth temperature (Akaji *et al.*, 2019). In the current study, the temperature of maximum leaf RGR was higher in *B. gymnorrhiza* (34.5 °C) than in *R. stylosa* (29.3 °C). These results suggest that the leaves of *B. gymnorrhiza* are more evolutionarily adapted to high growth temperature than *R. stylosa* leaves. The slight increase in the CC and C content of *B. gymnorrhiza* leaves at 30 °C growth temperature may also indicate better adaptation of this species. In some deciduous trees, an increase in the leaf content of lignin, which contains high amounts of C, helps to adjust to water stress under high light intensity (Niinemets and Kull, 1998).

### Response of respiratory costs to growth temperatures in two mangrove species

Although we observed an increase in RGR with growth temperature in both species, large proportions of leaf and root O_2_ respiration were used for maintenance at all growth temperatures examined (Fig. 8). The increase in O_2_ respiration rates as the growth temperature increased was due mainly to the increase in R_m_. We found that specific rates of root O_2_ respiration and NNUR were similar between *B. gymnorrhiza* and *R. stylosa* (Figs 1 and 6). Although specific rates of root ATP production and N uptake did not differ between the two species, *B. gymnorrhiza* had higher RMR and root RGR than *R. stylosa*. Thus, *B. gymnorrhiza* roots absorbed a lot of N at the whole-plant level, especially at above 20 °C growth temperature. The higher N uptake at the whole-plant level could be related to the higher Nc and leaf N content in *B. gymnorrhiza* than in *R. stylosa*. The higher leaf N content in *B. gymnorrhiza* indicates that the leaves of this species contained higher amounts of proteins to maintain; this could be one explanation for the high leaf R_m_ in *B. gymnorrhiza* at the high growth temperature. The leaf R_m_ and root R_m_ values of the two mangrove species were mostly within the range reported for temperate terrestrial plants, 2.0–15.6 nmol O_2_ g d. wt^−1^ s^−1^ (Zha *et al.*, 2001; van Iersel, 2006; Laureano *et al*., 2008; 2013; Hernández-Montes *et al.*, 2019), and thus the two mangroves do not require high production of respiratory ATP for maintenance even at high growth temperatures.

In this study, we estimated the potential O_2_ respiration rate for root N uptake, R_u_, by using a theoretical equation. The assumed *c*_u_ and estimated R_u_ strongly depend on the nitrate efflux (*E*) and electron partitioning to the AP (*τ*_a_) in roots [eqns (5) and (6)]. The ratio of *E* to nitrate influx (*I*) is 0.2–0.6 in other plant species (Bouma *et al.*, 1996; Scheurwater *et al.*, 1999; Ter Steege *et al.*, 1999), and can differ among plant species (Scheurwater *et al.*, 1999) and growth temperatures (Macduff *et al.*, 1994). We used a constant for the ratio of *E* to *I*, but the ratio may change depending on the growth temperatures in the two mangrove species. Regarding electron partitioning to the AP (*τ*_a_), the amounts of alternative oxidase (AOX) are influenced by growth temperature in various plant species (González-Meler *et al*., 1999; Kurimoto *et al.*, 2004*b*; Fiorani *et al.*, 2005; Watanabe *et al.*, 2008; Umbach *et al.*, 2009) and *τ*_a_ is affected by growth temperature in the leaves of *Populus canadensis* Moench (Searle and Turnbull, 2011) and *Quercus rubra* L. (Searle *et al.*, 2011*a*). In many cases, electron partitioning to the AP is less than to the CP (20/6 < P/O_2_ < 29/6) (Ribas-Carbo *et al.*, 1995; Millar *et al.*, 1998; Guy and Vanlerberghe, 2005; Armstrong *et al.*, 2008; Macfarlane *et al.*, 2009; Searle *et al*., 2011*a*, *b*). We assumed that *c*_u_ did not change with growth temperature or plant species in this study, but we cannot exclude that it does change. Based on the ratios of *E* to *I* and *τ*_a_ in previous studies, the actual R_u_ values of the two mangrove species may be in the range between the results of Fig. 8A and B. Further experiments to measure the nitrate efflux and *τ*_a_ dynamics of the mangroves at different growth temperatures will be needed to reveal the detailed R_u_ dynamics.

### CONCLUSIONS

We analysed the responses of growth parameters to growth temperature in the context of respiration cost for maintenance and growth in seedlings of two mangrove species and found that both species require a threshold temperature (12.2 °C in *B. gymnorrhiza* and 18.1 °C in *R. stylosa*) to ensure an O_2_ respiration rate sufficient for their leaf and root maintenance and growth. The link between leaf and root growth parameters and O_2_ respiration in the two mangrove species indicates that the underground temperature probably limits their growth under the low-temperature condition. Responses of whole-plant RGR to growth temperature showed that *B. gymnorrhiza* had the potential to adapt to a wider habitat temperature range than *R. stylosa*, which may be one explanation for the fact that *B. gymnorrhiza* grows in a wider range of western Indo-Pacific habitats than does *R. stylosa* (Spalding *et al.*, 2010; Supplementary data Fig. S1). In native habitats, *B. gymnorrhiza* tends to be on inland waterways, and *R. stylosa* tends to grow by the seaside. *Bruguiera gymnorrhiza* often grow as juveniles in shaded environments underneath their mother tree canopies. Therefore, compared with *B. gymnorrhiza* seedlings, the seedlings of *R. stylosa* may be more exposed to strong irradiance with high temperatures in the daytime. This could also explain the narrow distribution range of *R. stylosa* on the global scale because the region in which the air temperature does not exceed the limiting temperature for the mangrove growth may be narrower at the seaside than inland. Our results suggest that the effects of global warming caused by climate change could differ between the two mangrove species. In the future, other factors controlling the warm habitat range of mangroves should be examined; for example, mangrove plants grow in tidal flats where salinity stress can affect respiratory cost for maintenance and growth. Some studies have shown that salinity stress can affect RGR and respiration rates in mangrove species, but the magnitudes of these effects vary greatly among studies, among species and among organs (Burchett *et al.*, 1989; Lin and Sternberg, 1993; Lόpez-Hoffman *et al.*, 2006; Atreya and Bharagava, 2008; Paliyavuth *et al.*, 2009). Due to tidal export of organic matters from the soil, the levels of nutrients such as N and phosphorus (P) are low in tidal flats (Alongi *et al.*, 1992). Lovelock *et al.* (2006) observed an increase in the fine root respiration rate of another mangrove species, *Rhizophora mangle* L., following extra input of N and P. Examination of respiratory costs at high salinity and low N and P will further reveal the acclimation and adaptation mechanisms of these mangrove species.

## SUPPLEMENTARY DATA

Supplementary data are available online at https://academic.oup.com/aob and consist of the following. Table S1: *F*-values and *P*-values of two-way ANOVA of the effects of outside weather conditions and chamber on the chamber conditions. Table S2: *F*-values and *P*-values of one-way ANOVA of the effect of position on the daily accumulated air temperature, daily mean humidity and daily accumulated light intensity within a growth chamber. Table S3: *F*-values and *P*-values of two-way ANOVA for RGR, NAR, LAR, NNUR, LMR, RMR, LMA and Nc, and multifactor ANOVA for respiration rate, regression coefficient for growth, construction cost, protein content, C content, N content, nitrate N content and mineral content. Table S4: comparison of growth and respiration parameters in two mangrove species, *Bruguiera gymnorrhiza* and *Rhizophora stylosa* by Tukey’s multiple comparison tests. Table S5: estimated parameters of the models of the growth temperature dependencies of the growth and respiration parameters of two mangrove species, *Bruguiera gymnorrhiza* and *Rhizophora stylosa*. Figure S1: global distribution map and estimated range of air temperature of two mangrove species, *Bruguiera gymnorrhiza* and *Rhizophora stylosa*. Figure S2: growth chambers used for the cultivation experiment. Figure S3: profiles of air temperature, humidity and light intensity in the growth chambers. Figure S4: relationship between air temperature in the growth chambers and solar radiation and outside air temperature. Figure S5: estimated air temperatures in the chambers during the 56 d cultivation period. Figure S6: relationship between temperatures at leaf positions and at depths of 10 cm in soils. Figure S7: contents of nitrate N and minerals in leaves and roots of *B. gymnorrhiza* and *R. stylosa*.

mcab117_suppl_Supplementary_Data_S1

mcab117_suppl_Supplementary_Data_S2

mcab117_suppl_Supplementary_Data_S3

mcab117_suppl_Supplementary_Data_S4

mcab117_suppl_Supplementary_Data_S5

mcab117_suppl_Supplementary_Data_S6

mcab117_suppl_Supplementary_Data_S7

## References

[CIT0001] Abramoff MD, Magalhaes PJ, Ram SJ. 2004. Image processing with ImageJ. Biophotonics International 11: 36–42.

[CIT0002] Akaji Y, Inoue T, Tomimatsu H, Kawanishi A. 2019. Photosynthesis, respiration, and growth patterns of *Rhizophora stylosa* seedlings in relation to growth temperature. Trees 33: 1041–1049.

[CIT0003] Alongi DM, Boto KG, Robertson AI. 1992. Nitrogen and phosphorus cycles. In: Robertson AI, Alongi DM, eds. Tropical mangrove ecosystems. Washington, DC: American Geophysical Union, 251–292.

[CIT0004] Amthor JS . 1989. Respiration and crop productivity. New York: Springer-Verlag.

[CIT0005] Amthor JS . 1994. Respiration and carbon assimilate use. In: Boote KJ, Bennett JM, Sinclair TR, Paulsen GM, eds. Physiology and determination of crop yield. Madison, WI: American Society of Agronomy, 221–250.

[CIT0006] Armstrong AF, Badger MR, Day DA, et al 2008. Dynamic changes in the mitochondria electron transport chain underpinning cold acclimation of leaf respiration. Plant, Cell & Environment 31: 1156–1169. 10.1111/j.1365-3040.2008.01830.x 18507806

[CIT0007] Atkin OK, Bloomfield KJ, Reich PB, et al. 2015. Global variability in leaf respiration in relation to climate, plant functional types and leaf traits. New Phytologist 206: 614–636. 25581061 10.1111/nph.13253

[CIT0008] Atreya A, Bhargava S. 2008. Salt-induced respiration in *Bruguiera cylindrica* – role in salt transport and protection against oxidative damage. Physiology and Molecular Biology of Plants 14: 217–226. 23572889 10.1007/s12298-008-0021-3 PMC3550618

[CIT0009] Ball MC, Cochrane MJ, Rawson HM. 1997. Growth and water use of the mangroves *Rhizophora apiculata* and *R. stylosa* in response to salinity and humidity under ambient and elevated concentrations of atmospheric CO_2_. Plant, Cell & Environment 20: 1158–1166.

[CIT0010] Bouma TJ, Broekhuysen AGM, Veen BW. 1996. Analysis of root respiration of *Solanum tuberosum* as related to growth, ion uptake and maintenance of biomass. Plant Physiology and Biochemistry 34: 795–806.

[CIT0011] Brown JM, Outred HA, Hill CF. 1969. Respiratory metabolism in mangrove seedlings. Plant Physiology 44: 287–294. 16657058 10.1104/pp.44.2.287 PMC396077

[CIT0012] Bruhn D, Egerton JJG, Loveys BR, Ball MC. 2007. Evergreen leaf respiration acclimates to long-term nocturnal warming under field conditions. Global Change Biology 13: 1216–1223.

[CIT0013] Burchett MD, Field CD, Pulkownik A. 1984. Salinity, growth and root respiration in the grey mangrove, *Avicennia marina*. Physiologia Plantarum 60: 113–118.

[CIT0014] Burchett MD, Clarke CJ, Field CD, Pulkownik A. 1989. Growth and respiration in two mangrove species at a range of salinities. Physiologia Plantarum 75: 299–303.

[CIT0015] Cataldo DA, Haroon M, Schrader LE, Youngs VL. 1975. Rapid colorimetric determination of nitrate in plant-tissue by nitration of salicylic-acid. Communications in Soil Science and Plant Analysis 6: 71–80.

[CIT0016] Chapin FS . 1974. Morphological and physiological mechanisms of temperature compensation in phosphate absorption along a latitudinal gradient. Ecology 55: 1180–1198.

[CIT0017] Chapin FS, Bloom A. 1976. Phosphate absorption: adaptation of tundra graminoids to a low temperature, low phosphorous environment. Oikos 26: 111–121.

[CIT0018] Chapman VJ . 1962. Respiration studies of mangrove seedlings II. Respiration in air. Bulletin of Marine Science 12: 245–263.

[CIT0019] Clarkson TD, Jones LHP, Purves JV. 1992. Absorption of nitrate and ammonium ions by *Lolium perenne* from flowing solution cultures at low root temperatures. Plant, Cell & Environment 15: 99–106.

[CIT0020] Cornelissen JHC, Castro-Dίez P, Hunt R. 1996. Seedling growth, allocation and leaf attributes in a wide range of woody plant species and types. Journal of Ecology 84: 755–765.

[CIT0021] Crawford NM, Glass ADM. 1998. Molecular and physiological aspects of nitrate uptake in plants. Trends in Plant Science 3: 389–395.

[CIT0022] Duke NC, Ball MC, Ellison JC. 1998. Factors influencing biodiversity and distributional gradients in mangroves. Global Ecology and Biogeography Letters 7: 27–47.

[CIT0023] Ellison AM, Farnsworth EJ. 1997. Simulated sea level change alters anatomy, physiology, growth, and reproduction of red mangrove (*Rhizophora mangle* L.). Oecologia 112: 435–446. 28307619 10.1007/s004420050330

[CIT0024] Farnsworth EJ, Ellison AM, Gong WK. 1996. Elevated CO_2_ alters anatomy, physiology, growth, and reproduction of red mangrove (*Rhizophora mangle* L.). Oecologia 108: 599–609. 28307791 10.1007/BF00329032

[CIT0025] Fiorani F, Umbach AL, Siedow JN. 2005. The alternative oxidase of plant mitochondria is involved in the acclimation of shoot growth at low temperature. A study of *Arabidopsis AOX1a* transgenic plants. Plant Physiology 139: 1795–1805. 16299170 10.1104/pp.105.070789 PMC1310560

[CIT0026] Gonzalez-Meler MA, Ribas-Carbo M, Giles L, Siedow JN. 1999. The effect of growth and measurement temperature on the activity of the alternative respiratory pathway. Plant Physiology 120: 765–772. 10398711 10.1104/pp.120.3.765 PMC59314

[CIT0027] Guy RD, Vanlerberghe GC. 2005. Partitioning of respiratory electrons in the dark in leaves of transgenic tobacco with modified levels of alternative oxidase. Physiologia Plantarum 125: 171–180.

[CIT0028] Hachiya T, Noguchi K. 2008. Effect of growth temperature and total non-structural carbohydrate accumulation on growth coefficient in *Petunia*× *hybrida* petals. Physiologia Plantarum 134: 293–302. 10.1111/j.1399-3054.2008.01132.x 18494859

[CIT0029] Hernández-Montes E, Tomás M, Escalona JM, Bota J, Medrano H. 2019. Leaf growth rate and nitrogen content determine respiratory costs during leaf expansion in grapevines. Physiologia Plantarum 165: 746–754. 29885063 10.1111/ppl.12769

[CIT0030] Hothorn T, Bretz F, Westfall P. 2008. Simultaneous inference in general parametric models. Biometrical Journal 50, 346–363. 18481363 10.1002/bimj.200810425

[CIT0031] Hovenden MJ, Curran M, Cole MA, Goulter PFE, Skelton NJ, Allaway WG. 1995. Ventilation and respiration in roots of one-year-old seedlings of grey mangrove *Avicennia marina* (Forsk.) Vierh. Hydrobiologia 295: 23–29.

[CIT0032] van Iersel MW . 2006. Respiratory Q10 of marigold (*Tagetes patula*) in response to long-term temperature differences and its relationship to growth and maintenance respiration. Physiologia Plantarum 128: 289–301.

[CIT0033] Kurimoto K, Day DA, Lambers H, Noguchi K. 2004 *a*. Effect of respiratory homeostasis on plant growth in cultivars of wheat and rice. Plant, Cell & Environment 27: 853–862. 10.1093/pcp/pch116 15356327

[CIT0034] Kurimoto K, Millar AH, Lambers H, Day DA, Noguchi K. 2004 *b*. Maintenance of growth rate at low temperature in rice and wheat cultivars with a high degree of respiratory homeostasis is associated with a high efficiency of respiratory ATP production. Plant & Cell Physiology 45: 1015–1022. 15356327 10.1093/pcp/pch116

[CIT0035] Lambers H, Oliveira RS. 2019. Photosynthesis, respiration, and long-distance transport: respiration. In: Plant physiological ecology, 3rd edn. Cham: Springer Nature Switzerland AG, 115–172.

[CIT0036] Lambers H, Chapin FS III, Pons TL. 2008. Growth and allocation. In: Plant physiological ecology, 2nd edn. New York: Springer, 321–367.

[CIT0037] Laureano RG, Lazo YO, Linares JC, et al. 2008. The cost of stress resistance: construction and maintenance costs of leaves and roots in two populations of *Quercus ilex*. Tree Physiology 28: 1721–1728. 18765377 10.1093/treephys/28.11.1721

[CIT0038] Laureano RG, Gracίa-Nogales A, Seco JI, et al 2013. Growth and maintenance cost of leaves and roots in two populations of *Quercus ilex* native to distinct substrates. Plant and Soil 363: 87–99.

[CIT0039] Lin G, Sternberg LSL. 1993. Effects of salinity fluctuation on photosynthetic gas exchange and plant growth of the red mangrove (*Rhizophora mangle* L.). Journal of Experimental Botany 44: 9–16.

[CIT0040] Lόpez-Hoffman L, DeNoyer JL, Monroe IE, et al 2006. Mangrove seedling net photosynthesis growth and survivorship are interactively affected by salinity and light. Biotropica 38: 606–616.

[CIT0041] Lovelock CE, Ruess RW, Feller IC. 2006. Fine root respiration in the mangrove *Rhizophora mangle* over variation in forest stature and nutrient availability. Tree Physiology 26: 1601–1606. 17169899 10.1093/treephys/26.12.1601

[CIT0042] Macduff JH, Jarvis SC, Cockburn JE. 1994. Accumulation of NO_3_^–^ flux to low root temperature by *Brassica napus* in relation to NO_3_^–^ supply. Journal of Experimental Botany 45: 1045–1056.

[CIT0043] Macfarlane C, Hansen LD, Florez-Sarasa I, Ribas-Carbo M. 2009. Plant mitochondria electron partitioning is independent of short-term temperature changes. Plant, Cell & Environment 32: 585–591. 10.1111/j.1365-3040.2009.01953.x 19210639

[CIT0044] Martίnez F, Lazo YO, Fernández-Galliano RM, Marino JA. 2002 *a*. Chemical composition and construction cost for roots of Mediterranean trees, shrub species and grassland communities. Plant, Cell & Environment 25: 601–608.

[CIT0045] Martίnez F, Lazo YO, Fernández-Galliano RM, Marino JA. 2002 *b*. Root respiration and associated costs in deciduous and evergreen species of *Quercus*. Plant, Cell & Environment 25: 1271–1278.

[CIT0046] Millar AH, Atkin OK, Ian Menz R, Henry B, Farquhar G, Day DA. 1998. Analysis of respiratory chain regulation in roots of soybean seedlings. Plant Physiology 117: 1083–1093. 9662551 10.1104/pp.117.3.1083 PMC34924

[CIT0047] Niinemets Ü . 1999. Energy requirement for foliage formation is not constant along canopy light gradients in temperate deciduous trees. New Phytologist 141: 459–470.

[CIT0048] Niinemets U, Kull O. 1998. Stoichiometry of foliar carbon constituents varies along light gradients in temperate woody canopies: implications for foliage morphological plasticity. Tree Physiology 18: 467–479. 12651358 10.1093/treephys/18.7.467

[CIT0049] O’Leary BM, Asao S, Millar AH, Atkin OK. 2019. Core principles which explain variation in respiration across biological scales. New Phytologist 222: 670–686. 30394553 10.1111/nph.15576

[CIT0050] Osland MJ, Day RH, Larriviere JC, From AS. 2014. Aboveground allometric models for freeze-affected black mangroves (*Avicennia germinans*): equations for a climate sensitive mangrove–marsh ecotone. PLoS One 9: e99604. 24971938 10.1371/journal.pone.0099604 PMC4074035

[CIT0051] Osland MJ, Day RH, From AS, McCoy ML, McLeod JL, Kelleway JJ. 2015. Life stage influences the resistance and resilience of black mangrove forests to winter climate extremes. Ecosphere 6: article160.

[CIT0052] Osland MJ, Day RH, Hall CT, Brumfield MD, Dugas JL, Jones WR. 2017. Mangrove expansion and contraction at a poleward range limit: climate extremes and land–ocean temperature gradients. Ecology 98: 125–137. 27935029 10.1002/ecy.1625

[CIT0053] Paliyavuth C, Patanaponpaiboon P, Ninomiya I. 2009. Response of gas exchange characteristics and morphological features of two mangrove species, *Avicennia alba* and *Bruguiera gymnorrhiza*, to different salinity and light environments. Tropics 18: 23–33.

[CIT0054] Peterson GL . 1977. A simplification of the protein assay method of Lowry *et al*. which is more generally applicable. Analytical Biochemistry 83: 346–356. 603028 10.1016/0003-2697(77)90043-4

[CIT0055] Pickens CN, Hester MW. 2011. Temperature tolerance of early life history stages of black mangrove *Avicennia germinans*: implications for range expansion. Estuaries and Coasts 34: 824–830.

[CIT0056] Poorter H . 1994. Construction costs and payback time of biomass: a whole plants perspective. In: Roy J, Garnier E, eds. A whole plant perspective on carbon–nitrogen interactions. The Hague, the Netherlands: Academic Publishing, 111–127.

[CIT0057] Poorter H, Ganier E. 2007. Ecological significance of inherent variation in relative growth rate and its components. In: Pugnaire FI, Valladares F. eds. Functional plant ecology. Boca Raton, FL: CRC Press, 67–94.

[CIT0058] R Core Team . 2017. R: a language and environment for statistical computing. Vienna, Austria: R Foundation for Statistical Computing.

[CIT0059] Ribas-Carbo M, Berry JA, Yakir D, et al. 1995. Electron partitioning between the cytochrome and alternative pathways in plant mitochondria. Plant Physiology 109: 829–837. 12228636 10.1104/pp.109.3.829 PMC161383

[CIT0060] Saenger P, West PW. 2016. Determinants of some leaf characteristics of Australian mangroves. Botanical Journal of the Linnean Society 180: 530–541.

[CIT0061] Scheurwater I, Clarkson DT, Purves JV, et al 1999. Relatively large nitrate efflux can account for the high specific respiratory costs for nitrate transport in slow-growing grass species. Plant and Soil 215: 123–134.

[CIT0062] Searle SY, Turnbull MH. 2011. Seasonal variation of leaf respiration and the alternative pathway in field-grown *Populus* × *canadensis*. Physiologia Plantarum 141: 332–342. 21198649 10.1111/j.1399-3054.2010.01442.x

[CIT0063] Searle SY, Bitterman DS, Thomas S, Griffin KL, Atkin OK, Turnbull MH. 2011 *a*. Respiratory alternative oxidase responds to both low- and high-temperature stress in *Quercus rubra* leaves along an urban–rural gradient in New York. Functional Ecology 25: 1007–1017.

[CIT0064] Searle SY, Thomas S, Griffin KL, et al. 2011 *b*. Leaf respiration and alternative oxidase in field-grown alpine grasses respond to natural changes in temperature and light. New Phytologist 189: 1027–1039. 21128944 10.1111/j.1469-8137.2010.03557.x

[CIT0065] Spalding M, Kainuma M, Collins L. 2010. World atlas of mangroves. Washington, DC: Earthscan Ltd.

[CIT0066] Ter Steege MW, Stulen I, Wiersema PK, Posthumus F, Vaalburg W. 1999. Efficiency of nitrate uptake in spinach: impact of external nitrate concentration and relative growth rate on nitrate influx and efflux. Plant and Soil 208: 125–134.

[CIT0067] Thornley JHM, Johnson IR. 1990. Plant and crop modelling: a mathematical approach to plant and crop physiology. New York: Clarendon Press.

[CIT0068] Tjoelker MG, Oleksyn J, Reich PB. 1999. Acclimation of respiration to temperature and CO_2_ in seedlings of boreal tree species in relation to plant size and relative growth rate. Global Change Biology 5: 679–691.

[CIT0069] Tomlinson PB . 1986. Biogeography. In: Tomlinson PB, ed. The botany of mangroves. New York: Cambridge University Press, 40–61.

[CIT0070] Umbach AL, Lacey EP, Richter SJ. 2009. Temperature-sensitive alternative oxidase protein content and its relationship to flora reflectance in natural *Plantago lanceolata* populations. New Phytologist 181: 662–671. 19021863 10.1111/j.1469-8137.2008.02683.x

[CIT0071] Vertregt N, Penning de Vries FWT. 1987. A rapid method for determining the efficiency of biosynthesis of plant biomass. Journal of Theoretical Biology 128: 109–119.

[CIT0072] Walters MB, Kruger EL, Reich PB. 1993. Relative growth rate in relation to physiological and morphological traits for northern hardwood tree seedlings: species, light environment and ontogenetic considerations. Oecologia 96: 219–231. 28313418 10.1007/BF00317735

[CIT0073] Watanabe CK, Hachiya T, Terashima I, Noguchi K. 2008. The lack of alternative oxidase at low temperature leads to a disruption of the balance in carbon and nitrogen metabolism, and to an up-regulation of antioxidant defence systems in *Arabidopsis thaliana* leaves. Plant, Cell & Environment 31: 1190–1202. 10.1111/j.1365-3040.2008.01834.x 18507803

[CIT0074] White RE . 1972. Studies on mineral ion absorption by plants. I. The absorption and utilization of phosphorous by *Stylosanthes humilis*, *Phaseolus atropurpureus* and *Desmodium intortum*. Plant and Soil 36: 427–447.

[CIT0075] de Wit CT, Brouwer R, Penning de Vries FWT. 1970. The simulation of photosynthetic systems. In Šetlik I, ed. Prediction and measurement of photosynthetic productivity. Wageningen, the Netherlands: Pudoc, 47–70.

[CIT0076] Wright IJ, Reich PB, Atkin OK, Lusk CH, Tjoelker MG, Westoby M. 2005. Irradiance, temperature and rainfall influence leaf dark respiration in woody plants: evidence from comparisons across 20 sites. New Phytologist 169: 309–319. 10.1111/j.1469-8137.2005.01590.x 16411934

[CIT0077] Zha T, Ryyppö A, Wang K, Kellomäki S. 2001. Effects of elevated carbon dioxide concentration and temperature on needle growth, respiration and carbohydrate status in field-grown Scots pines during the needle expansion period. Tree Physiology 21: 1279–1287. 11696415 10.1093/treephys/21.17.1279

