# Correction to: Distinct responses of growth and respiration to growth temperatures in two mangrove species

**DOI:** 10.1093/aob/mcac090

**Published:** 2022-07-27

This is a correction to: Tomomi Inoue, Yasuaki Akaji, Ko Noguchi, Distinct responses of growth and respiration to growth temperatures in two mangrove species, *Annals of Botany*, Volume 129, Issue 1, 1 January 2022, Pages 15–28, https://doi.org/10.1093/aob/mcab117

In the originally published version of the article, “Distinct responses of growth and respiration to growth temperatures in two mangrove species” (doi: 10.1093/aob/mcab117), there was an error in equation (8). The correct form of this equation is:


$$\text{g}=\left(\text{CC}\times \frac{6}{180.15}\right)-\left(\frac{{\text{C}}_{\text{org}}}{12.01}\right)$$ (8)

This error has been corrected online.

