# Supplementary material for: Distinct responses of growth and respiration to growth temperatures in two mangrove species
Source: Ann Bot. 2021 Sep 11;129(1):15–28. doi: 10.1093/aob/mcab117 (PMC8752395; doi:10.1093/aob/mcab117)
Supplement: mcab117_suppl_Supplementary_Data_S1 [file mcab117_suppl_supplementary_data_s1.docx]

Supplementary data figure captions

Figure S1 Global distribution map and estimated range of air temperature of two mangrove species, *Bruguiera gymnorrhiza* and *Rhizophora stylosa*.

Figure S2 Growth chambers used for the cultivation experiment. (a) Outline of the design of the growth chambers. The control units for air temperature and humidity were positioned at the back side (north side) of the chambers, and the controlled air was supplied through holes in the floor. (b) The pots containing the experimental plants were positioned on 70-cm-high racks to prevent the wind from blowing directly against the pots. (c) Top view of a chamber. Temperature, humidity, and light intensity within each chamber were measured at three points located at 1 m from each other (yellow circles). Air temperature, humidity, and light intensity were measured with thermistor sensors (TR-72nw-S, T&D Corporation, Nagano, Japan), polymer thin-film sensors (TR-72nw-S, T&D Corporation), and quantum sensors (LI-190R, LI-COR, Nebraska, USA), respectively. All the sensors were calibrated and intercalibrated just before the measurements. Data for these parameters are shown in Fig. S3.

Figure S3 Profiles of (a, b) air temperature, (c–g) humidity, and (h–l) light intensity (PPFD) in the growth chambers. Variations in air temperature, humidity, and light intensity within the 20 °C chamber are shown in (b), (g), and (l), respectively; lines of different colours indicate different positions, which are located 1 m from each other in this chamber. Results of statistical analyses are shown in Tables S1 and S2.

Figure S4 Relationship between air temperature in the growth chambers and solar radiation and outside air temperature (a–c). Outside solar radiation and air temperature, which had been measured at a weather station located 0.7 km from the growth chambers, were obtained from the data archives of the Japan Meteorological Agency (<https://www.data.jma.go.jp/obd/stats/data/mdrr/index.html>). The air temperatures in the chambers were highly correlated with the outside solar radiation, but not with the outside air temperature (d). Because we did not have enough thermometers for parallel monitoring in the four growth chambers during the 56-day cultivation period, we used the regression equations in (d) to estimate the air temperatures in the chambers during the cultivation period (Supplementary Data Fig. S5).

Figure S5 Estimated air temperatures in the chambers during the 56-day cultivation period. The estimated values were calculated from the profiles of outside solar radiation during the cultivation period (obtained from the data archives of the Japan Meteorological Agency: <https://www.data.jma.go.jp/obd/stats/data/mdrr/index.html>) and the regression equations of the air temperature of the chambers against the outside solar radiation in Supplementary Data Fig. S4d.

Figure S6 Relationship between temperatures at leaf positions and at depths of 10 cm in soils. Blue, green, yellow, and red indicate data in the 15 °C-, 20 °C-, 25 °C-, and 30 °C-chamber, respectively. Regression equations are shown in the corresponding colours. Air and soil temperatures were not significantly different from each other (one-way ANOVA for all chambers combined, *F* = 0.01, *P* = 0.912).

Figure S7 Contents of (a, b) nitrate N and (c, d) minerals in (a, c) leaves and (b, d) roots of *B. gymnorrhiza* and *R. stylosa*. For other details, see the legend of Fig. 1.
