# Supplementary material for: Distinct responses of growth and respiration to growth temperatures in two mangrove species
Source: Ann Bot. 2021 Sep 11;129(1):15–28. doi: 10.1093/aob/mcab117 (PMC8752395; doi:10.1093/aob/mcab117)
Supplement: mcab117_suppl_Supplementary_Data_S7 [file mcab117_suppl_supplementary_data_s7.pptx]

## Slide 1
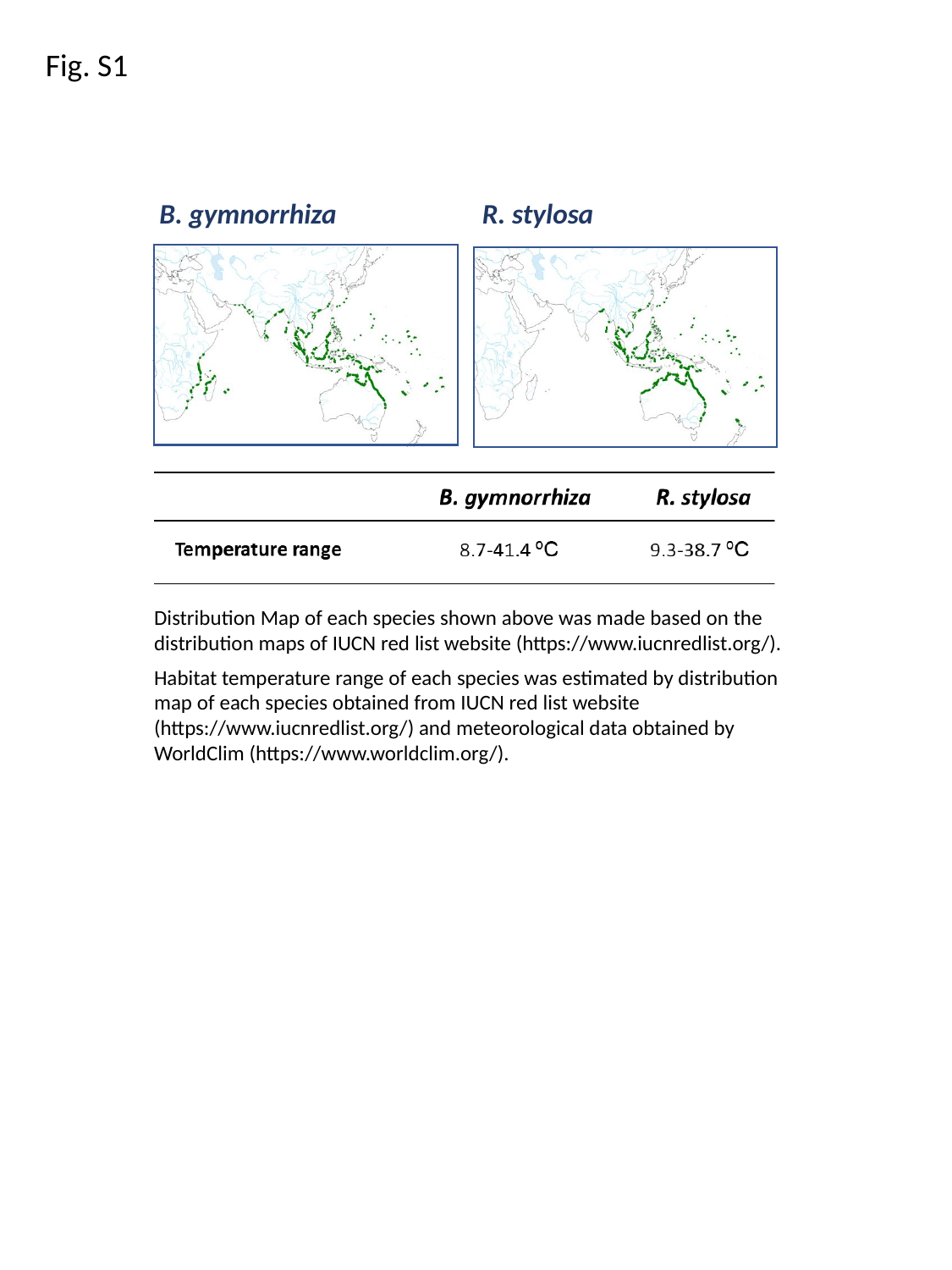

Fig. S1
B. gymnorrhiza
R. stylosa
Distribution Map of each species shown above was made based on the distribution maps of IUCN red list website (https://www.iucnredlist.org/).
Habitat temperature range of each species was estimated by distribution map of each species obtained from IUCN red list website (https://www.iucnredlist.org/) and meteorological data obtained by WorldClim (https://www.worldclim.org/).

## Slide 2
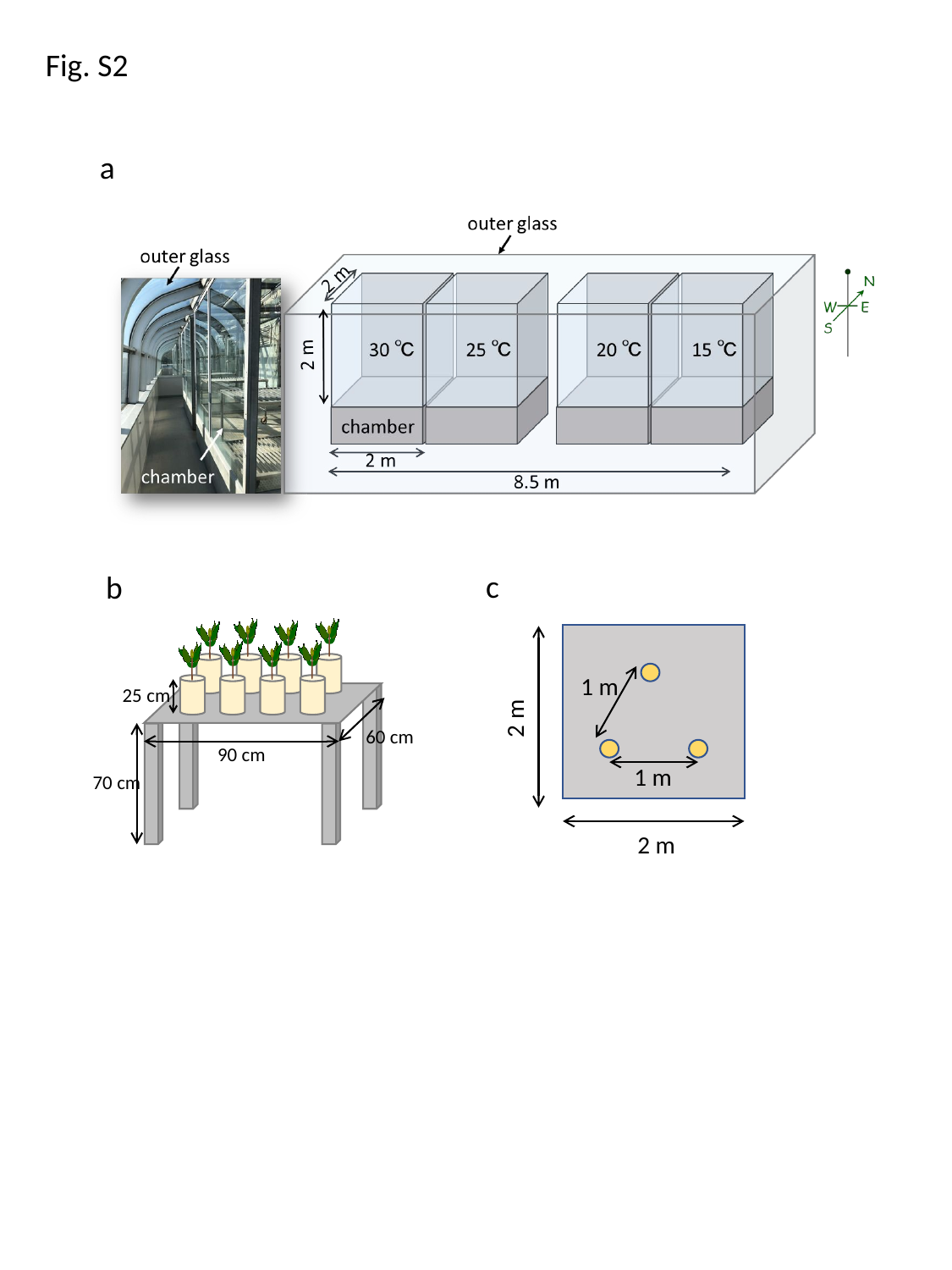

Fig. S2
a
c
b
25 cm
60 cm
90 cm
70 cm
1 m
2 m
1 m
2 m

## Slide 3
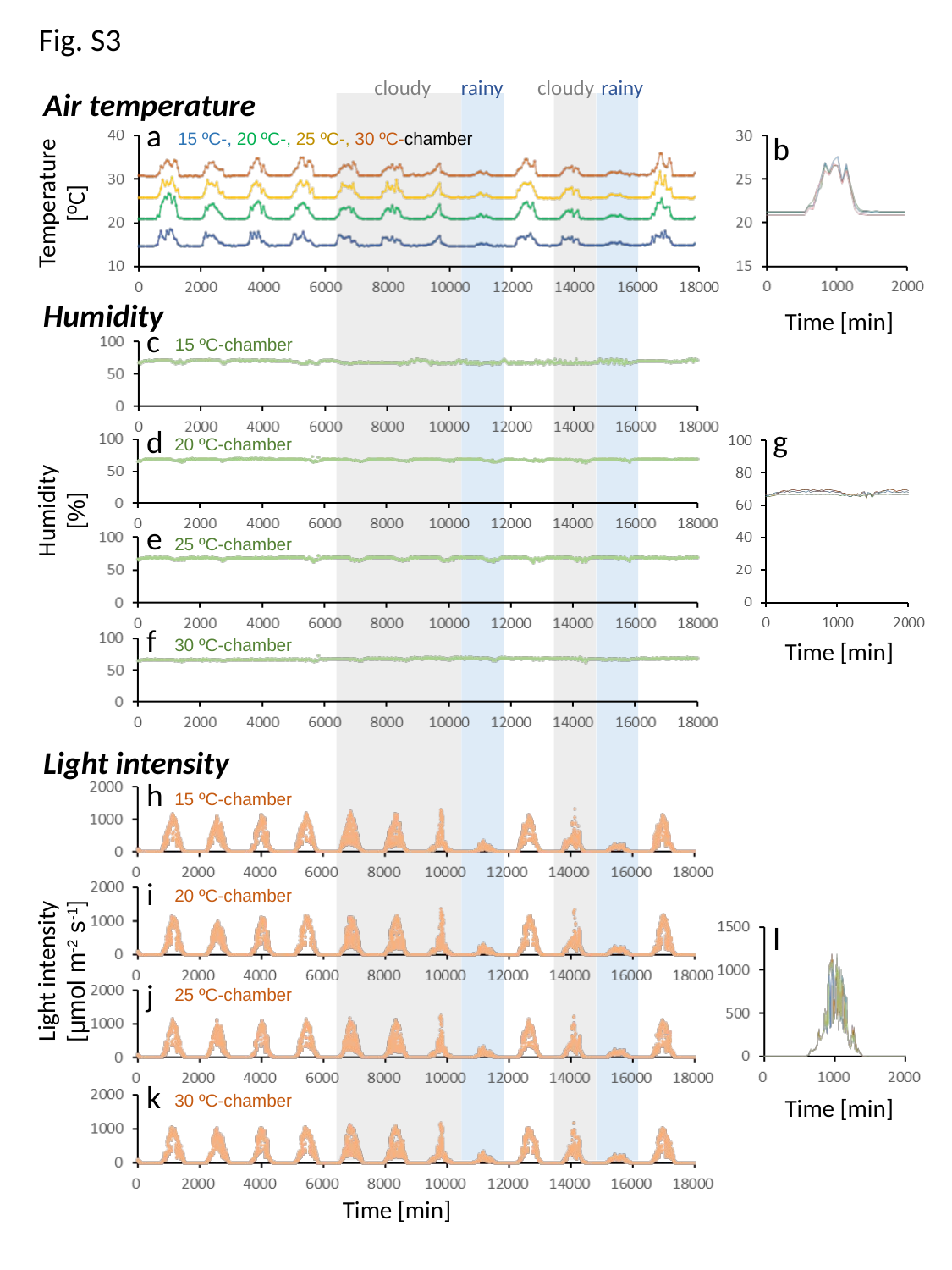

Fig. S3
cloudy
rainy
cloudy
rainy
Air temperature
a
15 ºC-, 20 ºC-, 25 ºC-, 30 ºC-chamber
b
Temperature
[ºC]
Humidity
Time [min]
c
15 ºC-chamber
g
d
20 ºC-chamber
Humidity
[%]
e
25 ºC-chamber
f
30 ºC-chamber
Time [min]
Light intensity
h
15 ºC-chamber
i
20 ºC-chamber
l
Light intensity
[μmol m-2 s-1]
j
25 ºC-chamber
k
30 ºC-chamber
Time [min]
Time [min]

## Slide 4
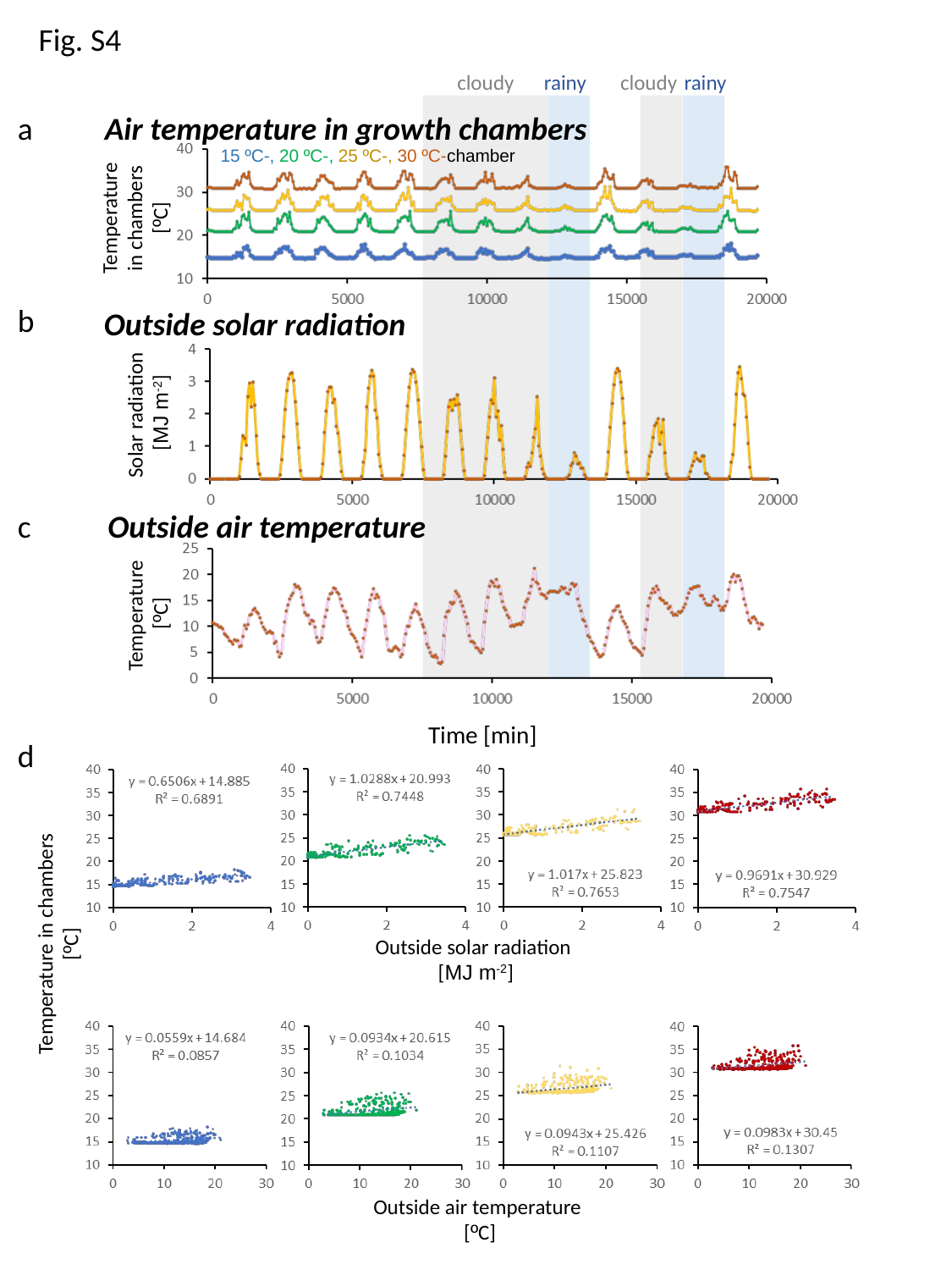

Fig. S4
cloudy
rainy
cloudy
rainy
a
Air temperature in growth chambers
15 ºC-, 20 ºC-, 25 ºC-, 30 ºC-chamber
Temperature
in chambers
[ºC]
b
Outside solar radiation
Solar radiation
[MJ m-2]
c
Outside air temperature
Temperature
[ºC]
Time [min]
d
Temperature in chambers
[ºC]
Outside solar radiation
[MJ m-2]
Outside air temperature
[ºC]

## Slide 5
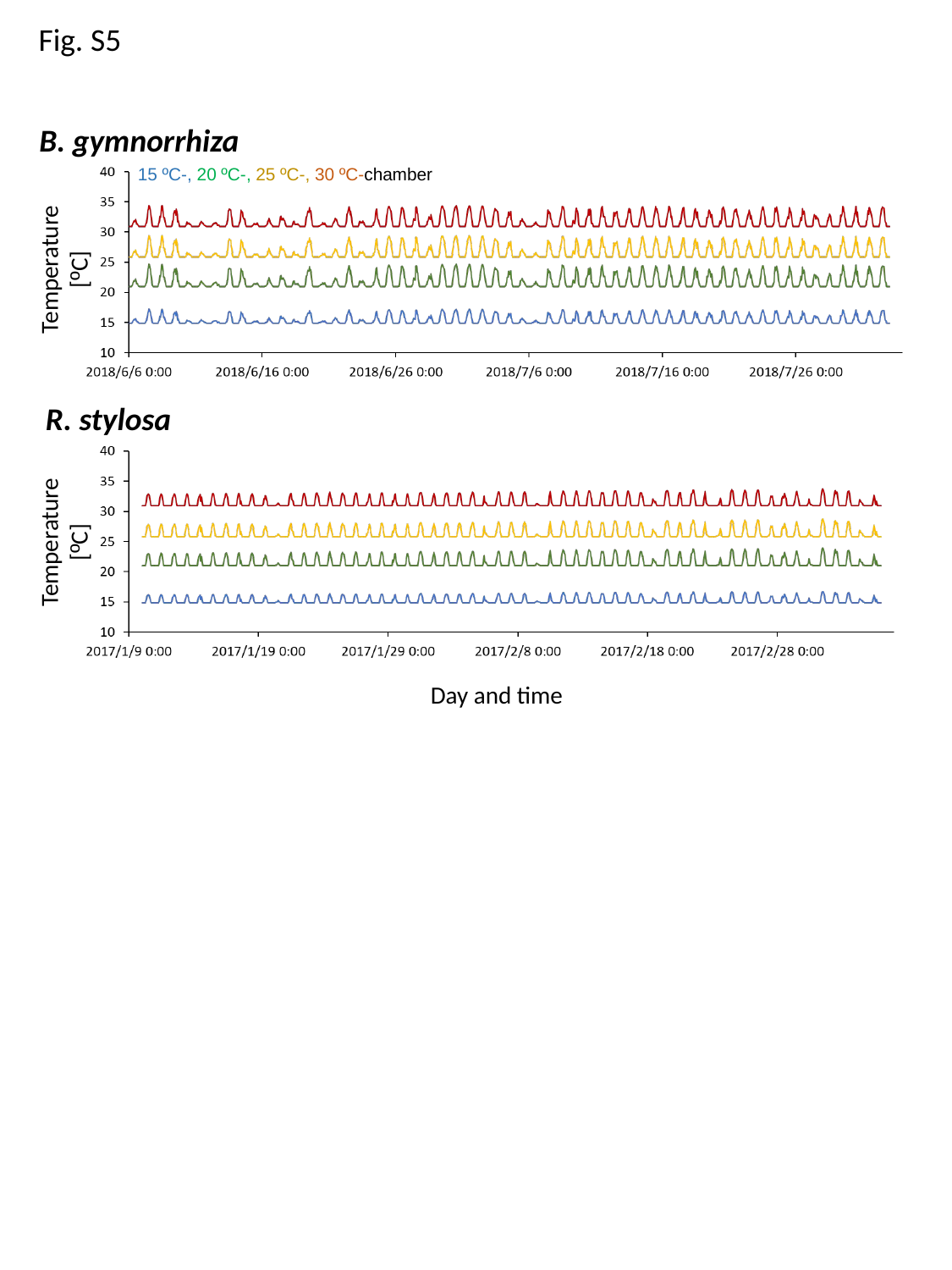

Fig. S5
B. gymnorrhiza
15 ºC-, 20 ºC-, 25 ºC-, 30 ºC-chamber
Temperature
[ºC]
R. stylosa
Temperature
[ºC]
Day and time

## Slide 6
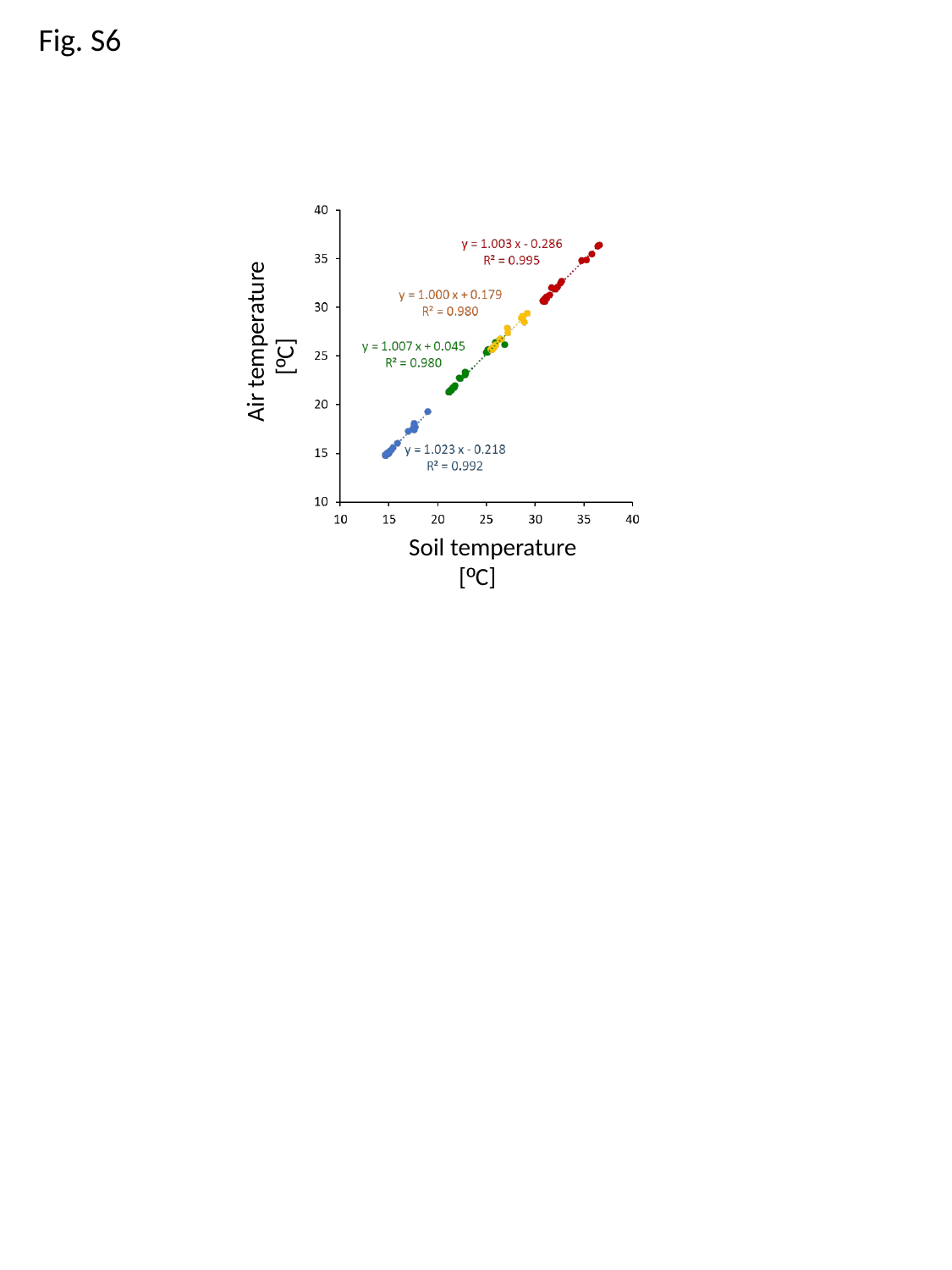

Fig. S6
　Air temperature
[ºC]
　Soil temperature
[ºC]

## Slide 7
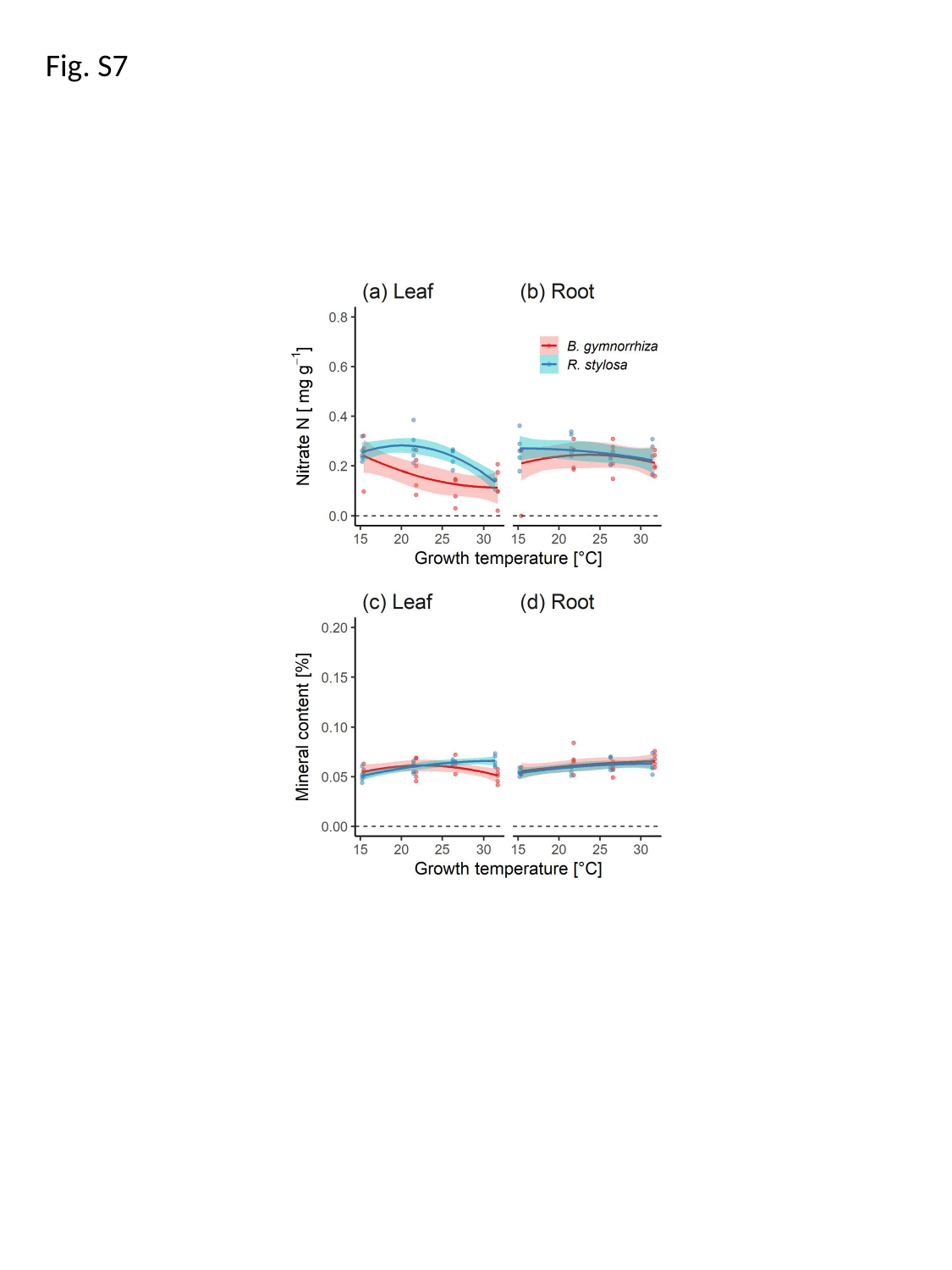

Fig. S7
